# Skeletons in the closet? Using a bibliometric lens to visualise phytochemical and pharmacological activities linked to *Sceletium*, a mood enhancer

**DOI:** 10.3389/fpls.2024.1268101

**Published:** 2024-03-21

**Authors:** Kaylan Reddy, Gary I. Stafford, Nokwanda P. Makunga

**Affiliations:** ^1^ Department of Botany and Zoology, Natural Sciences Faculty, Stellenbosch University, Stellenbosch, South Africa; ^2^ Department of Plant and Soil Sciences, University of Pretoria, Pretoria, South Africa

**Keywords:** alkaloid chemistry, central nervous system activity, Kanna, secondary metabolites, pharmacology, phytochemistry

## Abstract

Plants from the *Sceletium* genus (Aizoaceae) have been traditionally used for millennia by the Khoe and Khoen people in southern Africa, as an appetite suppressant as well as a mood elevator. In more recent times, this mood-elevating activity has been commercialised in the South African natural products industry for the treatment of anxiety and depression, with several products available both locally and abroad. Research on this species has seen rapid growth with advancements in analytical and pharmacological tools, in an effort to understand the composition and biological activity. The Web of Science (WoS) database was searched for articles related to ‘Sceletium’ and ‘Mesembrine’. These data were additionally analysed by bibliometric software (VOSviewer) to generate term maps and author associations. The thematic areas with the most citations were South African Traditional Medicine for mental health (110) and anxiolytic agents (75). Pioneer studies in the genus focused on chemical structural isolation, purification, and characterisation and techniques such as thin layer chromatography, liquid chromatography (HPLC, UPLC, and more recently, LC-MS), gas chromatography mass spectrometry (GC-MS), and nuclear magnetic resonance (NMR) to study mesembrine alkaloids. Different laboratories have used a diverse range of extraction and preanalytical methods that became routinely favoured in the analysis of the main metabolites (mesembrine, mesembranol, mesembranone, and Sceletium A4) in their respective experimental settings. In contrast with previous reviews, this paper identified gaps in the research field, being a lack of toxicology assays, a deficit of clinical assessments, too few bioavailability studies, and little to no investigation into the minor alkaloid groups found in *Sceletium*. Future studies are likely to see innovations in analytical techniques like leaf spray mass spectrometry and direct analysis in real-time ionisation coupled with high-resolution time-of-flight mass spectrometry (DART-HR-TOF-MS) for rapid alkaloid identification and quality control purposes. While *S. tortuosum* has been the primary focus, studying other *Sceletium* species may aid in establishing chemotaxonomic relationships and addressing challenges with species misidentification. This research can benefit the nutraceutical industry and conservation efforts for the entire genus. At present, little to no pharmacological information is available in terms of the molecular physiological effects of mesembrine alkaloids in medical clinical settings. Research in these fields is expected to increase due to the growing interest in *S. tortuosum* as a herbal supplement and the potential development of mesembrine alkaloids into pharmaceutical drugs.

## Introduction

**Figure 1:**
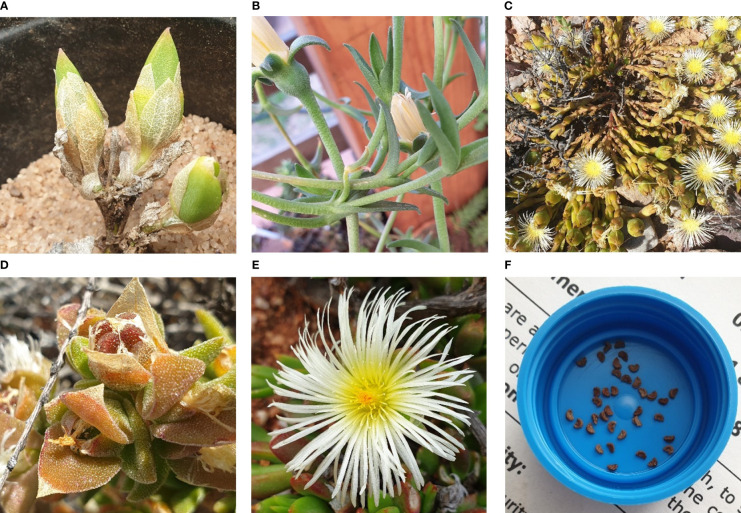
**(A)** *Sceletium rigidum*; **(B)** Image of *Sceletium subvelutium* (syn. *Mesembryanthemum varians*); **(C)** climbing or decumbent habit form of growth; **(D)** characteristic idioblasts (bladder-like cells) on *Sceletium* leaves; **(E)** flower structure of *Sceletium* species and **(F)** characteristic kidney-shaped seeds. (All images taken by N Makunga and K Reddy).

The plant *Mesembryanthemum tortuosum* (syn. *Sceletium tortuosum*) (L.) N.E.Br. has well-documented medicinal activity and ethnopharmacology (Smith et al., 1998; Gericke and Viljoen, 2008) and is thus the most popular from the *Sceletium* genus (Family: Aizoaceae, subfamily: Mesembryanthemoideae). *S. tortuosum* is also referred to as kanna, channa, kougoed, or ‘sceletium’ (Smith et al., 1998). This species is a climbing or creeping perennial with succulent leaves and stems that become thick and slightly woody with age (Klak et al., 2007). An important diagnostic feature of this genus is the skeletonised veins that are apparent when leaves dry (**Figure 1A**). The typical growth form exhibits a scandent nature (**Figures 1B, C**) together with leaves that have idioblasts or ‘bladder cells’ (**Figure 1D**). The flower colour of petals ranges from white, yellow to pale pink (**Figure 1E**). The seeds of *Sceletium* species are brown to black kidney-shaped, and these are small in diameter ranging from 1 mm to 2 mm (**Figure 1F**).

**Figure 2:**
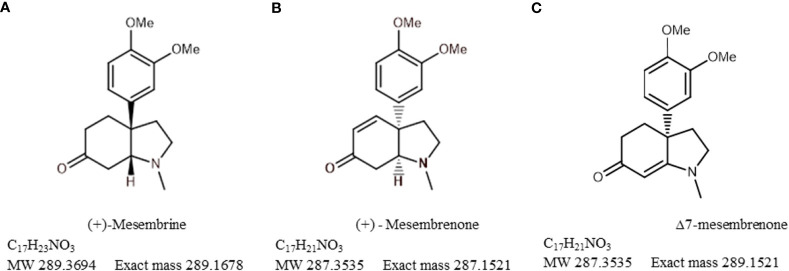
Chemical structures of **(A)** mesembrine, **(B)** mesembrenone, and **(C)** Δ^7^mesembrenone.

The plant is indigenous to southern Africa where it has been traditionally used in folk medicine by the Khoekhoen and Sān (Khoe-Sān/KhoiSan) people as a masticatory agent or as a mood elevator (Gericke and Viljoen, 2008). More recently, *S. tortuosum* has been commercialised as an antidepressant or anxiolytic and it is also recommended for attention-deficit disorders, as it aids in mental alertness (Harvey et al., 2011). The chemical constituents which were recognised for their medicinal activity are a group of mesembrine alkaloids that are uniquely associated with *Sceletium* species; however, they do share some similarities with Amaryllidaceae alkaloids. There has been a particular emphasis on mesembrine (**Figure 2A**), mesembrenone (**Figure 2B**), and Δ^7^mesembrenone (**Figure 2C**) as biomarker compounds due to more scientific information being available in terms of chemical characterisation and for commercial quality assurance profiling regimes. Thus far, there have been several comprehensive reviews based on the chemistry of alkaloids found in *Sceletium* (Jeffs et al., 1982; Lewis, 1995; 2001; Jin, 2016; Jin and Yao, 2019). Although this list may not necessarily be comprehensive as it is based on a Scopus database search, other reviews that focus on *Sceletium* and its phytochemistry and pharmacology include the work of Gericke and Viljoen (2008); Stafford et al. (2008); Van Wyk (2011); Van Wyk (2015); Krstenansky (2017); Makolo et al. (2019), and Faro et al. (2020). These reviews discuss 1) the ethnobotanical history and chemical diversity in the genus (Smith et al., 1998); 2) the pharmacological and chemical evidence of ethnobotanical use in *Sceletium* (Gericke and Viljoen, 2008); 3) plants from South Africa with CNS effects used for mental health purposes (Stafford et al., 2008); 4) the commercial potential of medicinal plants in South Africa (Van Wyk, 2011, 2015); 5) the occurrence, chemistry, and pharmacology of mesembrine alkaloids (Krstenansky, 2017); 6) the distribution, structural elucidation, biosynthesis, organic synthesis, chemotaxonomy, and biological activities of (−)-mesembrine from *Sceletium* species (Makolo et al., 2019); and 7) the biomedical activities of new psychoactive substances from natural origins (Faro et al., 2020). Within this current paper, we provide an update on analytical techniques used to study *Sceletium tortuosum* and its relatives, where possible. We also summarise studies that focus on chemical variation as much quantitative and qualitative information is still presently missing with regard to the biochemical components that make up the phytochemical profiles of these plants. This paper also presents findings on the use of VOSviewer to identify gaps and trends in *Sceletium* research, which may be of value for other scientists and industry to decide on areas to research within the available options. Furthermore, there is great interest in the use of *Sceletium* species and *Sceletium* alkaloids against anxiety (Shikanga et al., 2011; Loria et al., 2014) and depression (Gericke and Viljoen, 2008; Krstenansky, 2017) but preclinical and clinical evidence that validates these particular applications, which are grounded in an ethnobotanical context, is still limited. In spite of this, the commercialisation of *S. tortuosum* for various phyto-pharmaceutic markets is on the rise (Patnala and Kanfer, 2013; Krstenansky, 2017).

In order to get an overview of the available literature, a systematic bibliometric analysis was undertaken. Currently, there is a growing body of scientific literature that is based on chemical and pharmaceutical studies that have focused on *S. tortuosum* but recent studies on the taxonomy and geographical occurrence of the other *Sceletium* species are limited. This is of relevance as species misidentifications and biodiversity losses may prevail. The first part of this review thus aimed to collate information linked to the taxonomy and distribution of *Sceletium* species. These data were collected from databases such as SANBI-BODATSA and iNaturalist as an introduction before an update on the pharmacology and chemistry observed within the genus is presented. It is imperative to prioritise the correct collection of species, and as such an understanding of the taxonomy of the genus should be consulted. The current trends within the literature and associated authors on a global scale. The present review summarises the studies conducted on the *Sceletium* genus and its chemical constituents over time in terms of the progress in phytochemistry, ethnobotanical use, and pharmacology. This work intends to expose the current gaps within *Sceletium* research. Here, we report on studies from 1961 to the present and direct attention to recent advancements and future directions that may further develop quality, safety, and toxicological standards for therapeutic and nutraceutical applications concerning *S. tortuosum* and its relatives.

### Taxonomy and distribution

The species currently recognised are *S. crassicaule* (Haw.) L. Bolus, *S. emarcidum* (Thunb.) L. Bolus ex H.J. Jacobson, *S. exalatum* Gerbaulet, *S. expansum* (L.) L. Bolus, *S. rigidum* (**Figure 1A**), L. Bolus, *S. strictum* L. Bolus, *S. tortuosum*, and *S. varians* (Haw.) Gerbaulet (**Figure 1B**), as revised by Gerbaulet (Gerbaulet, 1996). Several species were reduced to being combined into the same species including *S. joubertii* L. Bol. and *S. namaquense* L. Bol., now considered to be part of the *S. tortuosum* complex. Taxonomically the plant genus was established in 1925 by N.E. Brown, but Klak et al. (2007), in their phylogenetic study of the family, proposed that Mesembryanthemoideae should consist of the single genus *Mesembryanthemum*. Thus, *Sceletium* was reduced to synonymy to *Mesembryanthemum*, and thus, the eight species of *Sceletium* (above) are currently accepted as *Mesembryanthemum crassicaule* Haw., *M. emarcidum* Thunb., *M. exalatum* (Gerbaulet) Klak*, M. expansum* L., *M. archeri* (L. Bolus) Klak (*=S. rigidum*), *M. ladismithiense* Klak (*=S. strictum*), *M. tortuosum* L., and *M. varians* Haw. However, for the purpose of this particular article, *Sceletium* is used as this is still predominantly used in industry, in scientific works on the commercially important *Sceletium tortuosum*, particularly related to its chemistry and pharmacology, and non-scientific settings. The conservation status of species within the *Sceletium* genus is also variable with several members of the genus being evaluated as threatened (*S. expansum*, *S. strictum*, and *S. varians*) by the South African National Biodiversity Institute’s Threatened Species Programme (http://redlist.sanbi.org/, **Table 1**). With *S. strictum*, being categorised as endangered (EN) and *S. expansum* and *S. varians*, both listed as vulnerable (VU). All other species in the genus are considered as being of least concern (LU).

**Table 1:** SANBI Red List conservation status of the species of the *Sceletium* genus.

Species	SANBI Red List conservation status
*Sceletium tortuosum* (L) N.E.Br	Least concern (LC)
*Sceletium varians* (Haw.) Gerbaulet	Vulnerable (VU)
*Sceletium strictum* L. Bolus	Endangered (EN)
*Sceletium rigidum* L. Bolus	Least concern (LC)
*Sceletium crassicaule* L. Bolus	Least concern (LC)
*Sceletium expansum* (L.) L. Bolus	Vulnerable (VU)
*Sceletium exaltum* L. Bolus Gerbaulet	Least concern (LC)
*Sceletium emarcidum* (Thunb.) L. Bolus ex H.Jacobsen	Least concern (LC)

**Figure 3:**
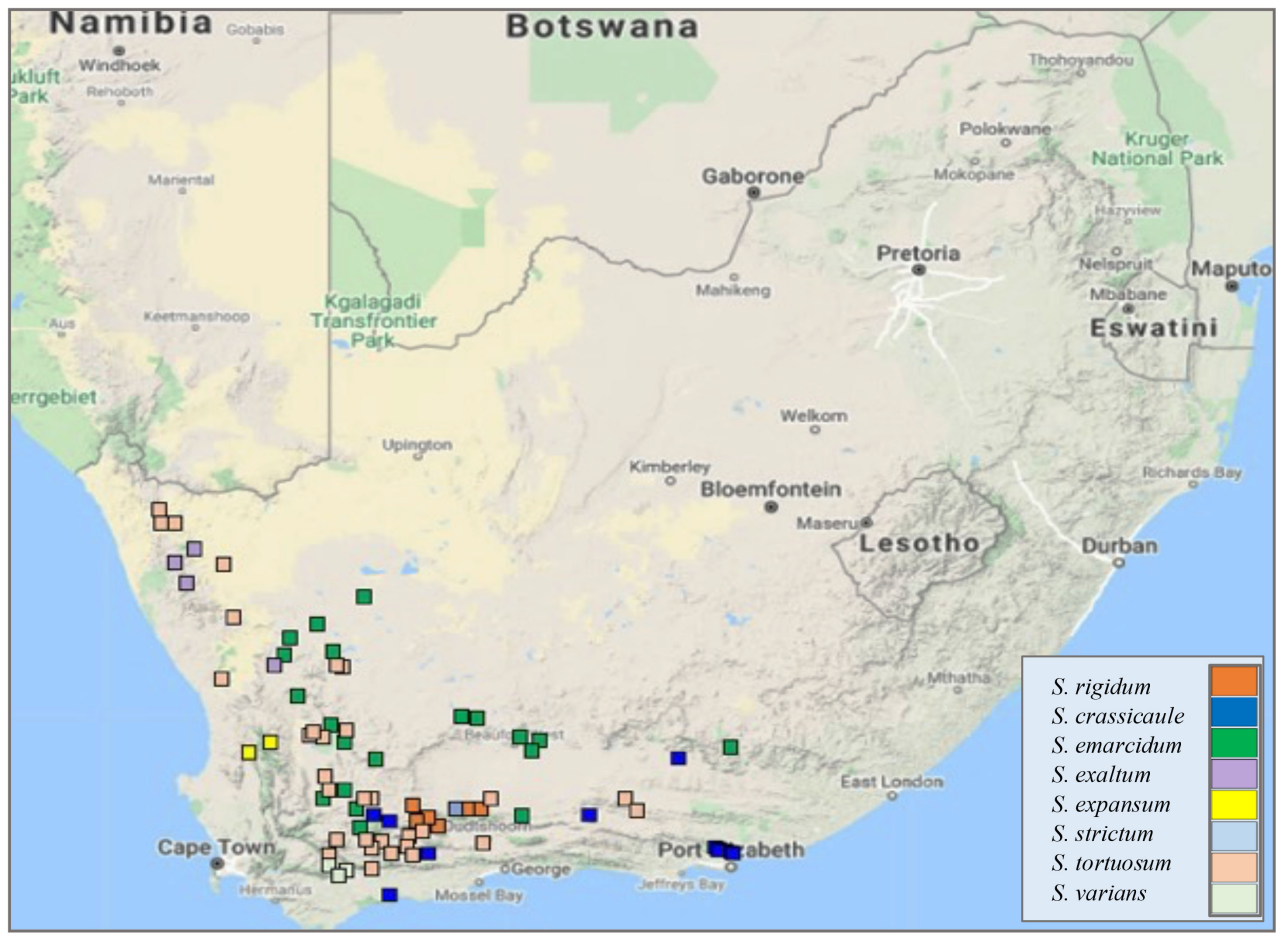
A Geographic distribution of wild collections of eight species of *Sceletium* in South Africa (data obtained from SANBI-BODATSA Database).

As part of this review, a distribution map of *Sceletium* species was generated from the SANBI-BODATSA (South African National Biodiversity Institute - Botanical Database of Southern Africa); this database contained information sourced from observational data, herbaria, literature, collector information, and species checklists. The majority of the observations were in the Western Cape of South Africa with some in the Northern and Eastern Cape provinces, as illustrated in **Figure 3**. A particular emphasis has been placed on *S. tortuosum* in the literature for its medicinal properties. The distribution of *S. tortuosum* has been reported in the southwestern areas of South Africa (Gericke and Viljoen, 2008). The plant has an affinity for arid environments and has been reported to grow from Namaqualand through to Aberdeen in South Africa (Chesselet, 2005).

### Ethnobotany

Simon van der Stel’s, the last commander and first Governor of the Dutch Cape Colony, journey to Coperbergh (near present-day Okiep and Carolusberg, in the Northern Cape, South Africa) in 1685 made note of how *kanna* was consumed by the native people, and details of its processing were included in the descriptions related to the species. The journal had the following quotation (translated from Dutch):

“They chew mostly a certain plant which they call Canna and which they bruise, roots as well as the stem, between the stones and store and preserve in sewn-up sheepskins”.

Between the date ranges of 1772 and 1774, a Swiss botanist and student of Linnaeus, Carl Peter Thunberg, made journeys to the Eastern Cape and reported on the value of the sedative plants that were found in the locality of present-day Oudtshoorn in the Little Karoo, South Africa (Gordon, 1996). Other reports followed: the plants were used as tinctures (Pappe, 1857), snuffed or smoked or as teas (Jacobson, 1960; Smith et al., 1996; Van Wyk and Wink, 2018), or recreationally (Hartwich and Zwicky, 1914). Watt and Breyer-Brandwijk (1962) indicated that in Namaqualand, both the aerial and underground (root) parts were used to make *kougoed* and how *Sceletium tortuosum* was used as an agent to help with pain, hunger relief, cholic, and restlessness in infants by the Nama people. Since the review paper of Smith et al. (1998), an increasing body of scientific information, associated in particular with *Sceletium tortuosum*, has emerged, leading to continuous progress in the areas of phytochemistry and pharmacology. This review aimed to provide visual networks linked to past research and identified current trends. We provide a historical account of the use of analytical techniques and pharmacological bioassays that have been employed to study *S. tortuosum* and its relatives. Finally, gaps in knowledge, recommendation, and best practice in studying these neurologically acting medicinal plants are presented.

## Method—bibliometric analysis

### Data sources

The Web of Science Core Collection (Clarivate Analytics, United States) was chosen as the data source. In August 2023, we conducted a search of the topic (phrases appearing in titles, abstracts, and keywords) using the following search terms: ‘Sceletium’ OR ‘mesembrine’ NOT ‘Gastropoda’. A bibliometric data analysis, for the period 1961–2023, was used to determine trends within previous investigations and how *Sceletium* research has evolved, through tracking patterns, trends, relationships, and the development of a discipline over time. Titles and abstracts were screened to exclude false-positives (papers that were not exclusively on *Sceletium* or mesembrine-type compounds found within the *Sceletium* genus). No supplementary restrictions had been placed on document type (review, editorial, letter, etc.) and assay model (*in vivo*, *in silico*, *in vitro*, etc.). The average citation amongst the most popular thematic areas within the body of knowledge associated with *Sceletium* is represented as a bar graph generated in Excel.

**Figure 4:**
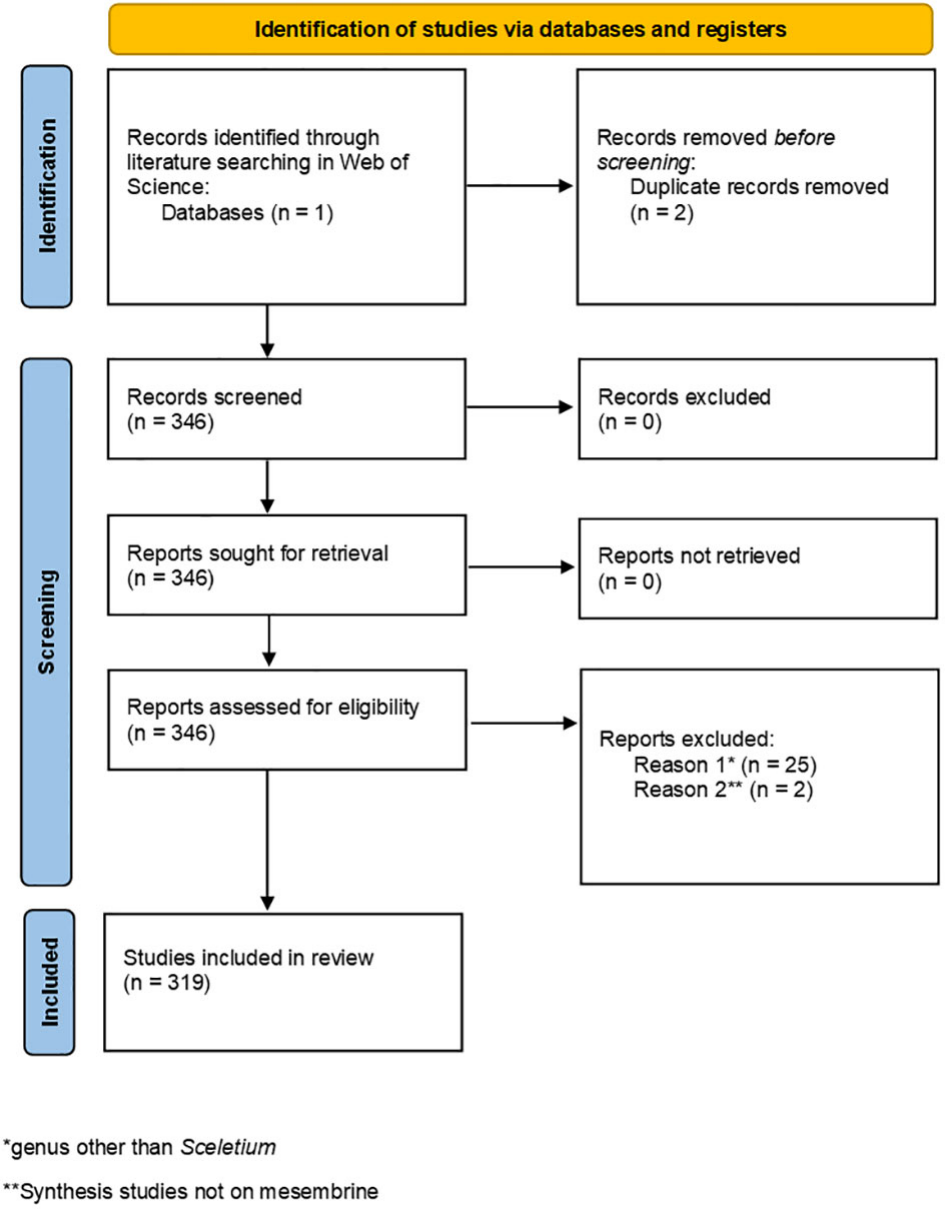
PRISMA chart illustrating the process used to screen studies for eligibility in this systematic review.

The literature search resulted in 348 articles being eligible for the systematic review, and two duplicate studies were removed (**Figure 4**). After reviewing the abstracts of 346 articles, 27 articles were removed on the basis of 25 being irrelevant due to the study either being focused on a different genus than *Sceletium* or studies investigating the occurrence of mesembrine in other species aside from *Sceletium.* Two additional organic synthesis studies were removed based on their contents not directly linking to mesembrine alkaloid synthesis. Finally, only 319 studies were included for analysis, as indicated in the PRISMA chart (**Figure 4**).

The data from our WOS searches were read from a bibliographic database file (i.e., the.txt file). Different types of analyses were performed based on our research questions. We were interested in determining the following: 1) the number of contributions in the field and how this changed with time; 2) authorship patterns; 3) geographical location of the producers of the articles, and finally; 4) identification of trends and gaps in the field.

### Term maps

Term maps were generated using words in the titles and abstracts whilst authorship and country maps were generated from information associated with the authors and affiliations. Within the bibliographic analysis, 319 articles were analysed and visualised by VOSviewer (Van Eck and Waltman, 2010). VOSviewer is a software that visualises patterns between authors, countries, and terms found in a body of literature. The software creates networks between the data and illustrate them as bubbles connected by lines, indicating association. The larger the bubble, the greater its frequency of occurrence. The thicker the lines the greater number of links an item has with others in the network. Irrelevant phrases or repetitions of phrases were excluded.

## Discussion

### Past and current trends in literature

From the 319 articles that were published on *Sceletium* and Mesembrine-type alkaloids from *Sceletium*, the document types were predominantly articles (n = 264) and reviews (n = 55). The citations received by the 319 articles in this domain ranged from 0 to 230 (mean ± SD = 26.02 ± 28.18). The most cited paper was between two papers, the first an ethnobotanical review by Stafford et al. (2008) investigating traditional South African plants with CNS activity (8.86 citations per year). This was followed by the Gu and You (2011) paper on the organic synthesis of mesembrine isomers (11.27 citations per year). The hundred most cited papers within the field had an average citation of 55, with an average yearly citation of 4.

**Figure 5:**
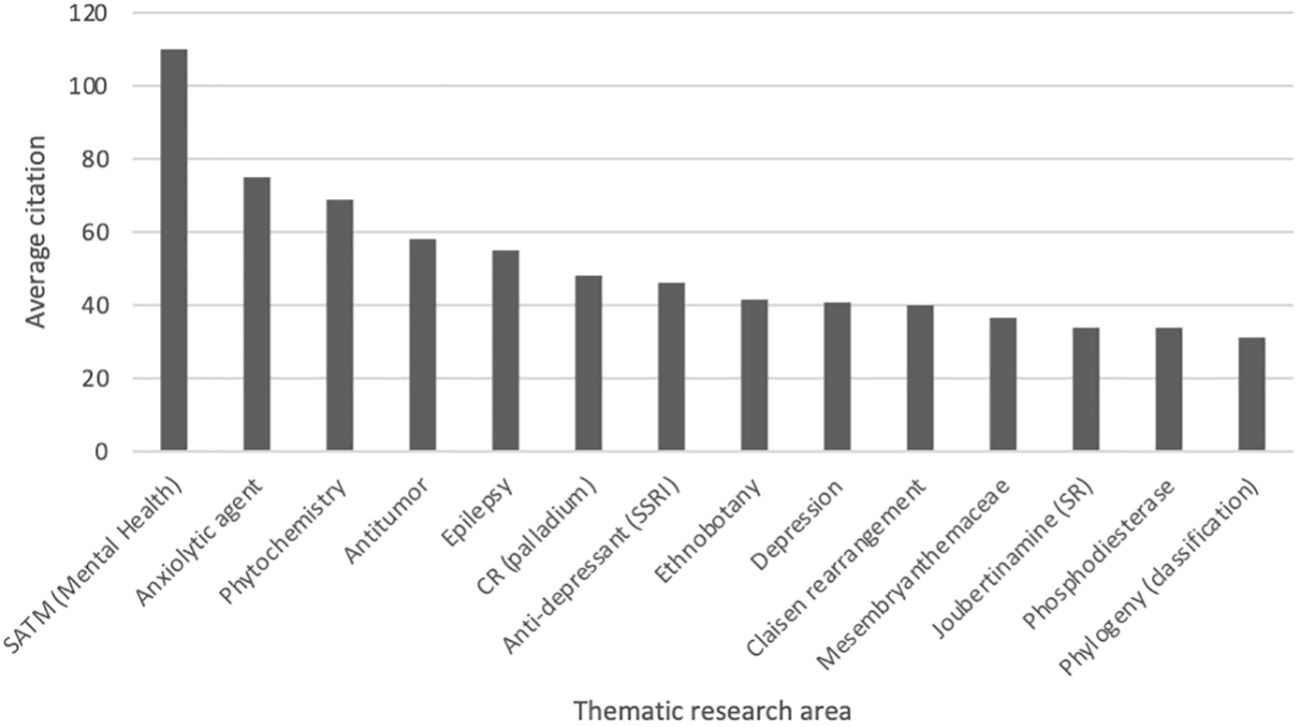
Average citation of thematic research areas within *Sceletium* research from Web of Science (n = 296, citation values >30.00). SATM (Mental health), South African Traditional Medicine (Mental Health); CR (palladium), Coupling reactions (palladium); Joubertiamine (SR), Joubertinamine (sigmatropic rearrangements).

The thematic areas where the majority of the research is focussed were as follows: Chemistry; Molecular Biology; and Pharmacology. The average citation amongst the most popular thematic areas associated with *Sceletium* research is presented in **Figure 5**.

For this reason, this review has a stronger emphasis on the work conducted in these fields. A particular focus has been placed on one species, *S. tortuosum* (119 links to other topics), and the membrane-rich extracts (64 links to other topics) of this plant. This has been the trend since the initial scientific interest in the plant in the 1960s. It is also interesting to note a lack of publications between 1980 and 2000. Dominant investigation areas were identified as ‘chemistry’ and ‘pharmacology’ especially, those focussing on *Sceletium* alkaloids to further understand the medicinal application of this plant (**Figure 5**).

### Key research themes

**Figure 6:**
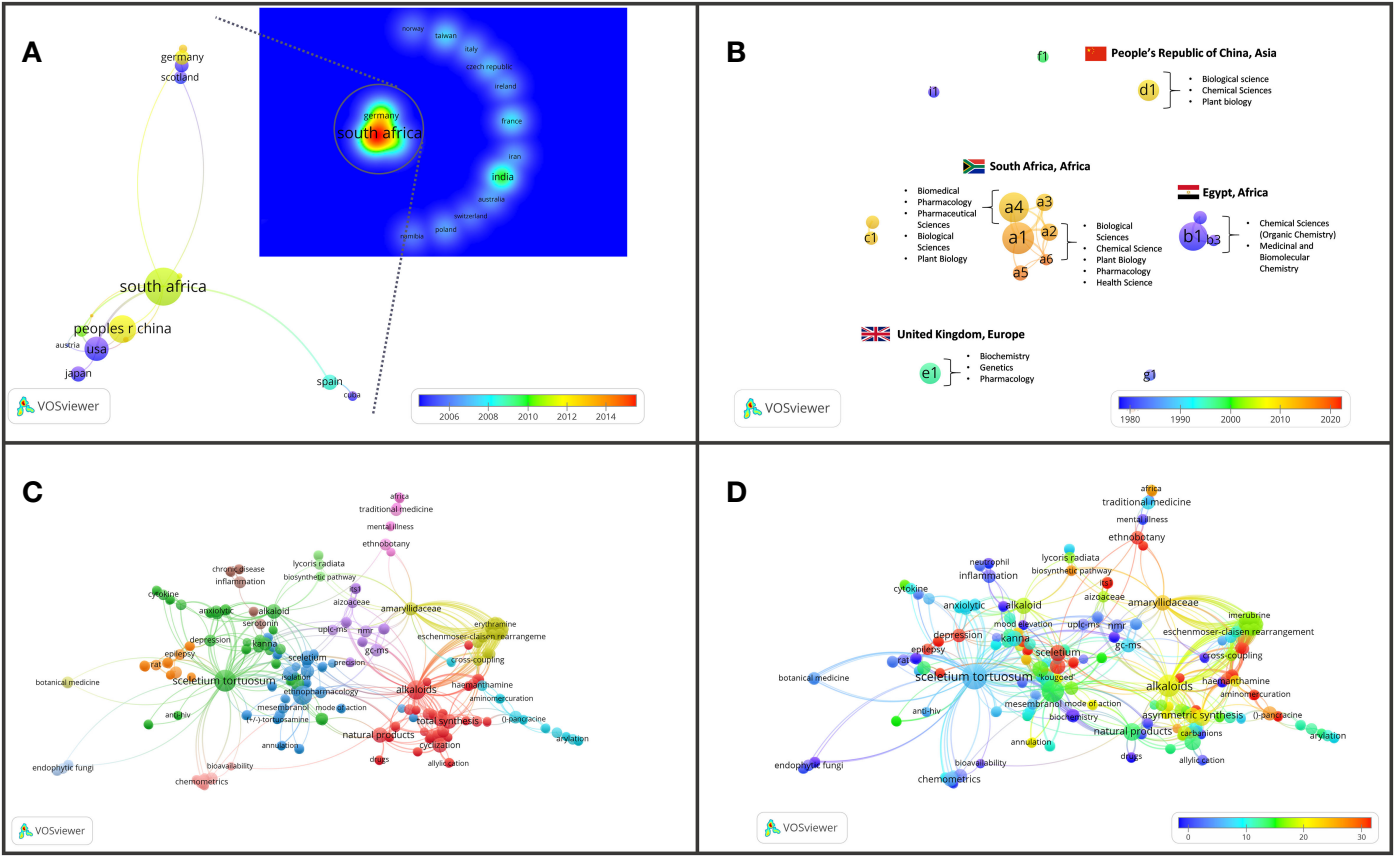
Major bibliographic summaries of literature in *Sceletium* research. **(A)** Country network map of the most prolific research network in *Sceletium* research, based on country affiliation. Image additionally illustrating Average publication year. **(B)** Author network map showing linkages and collaboration between various researchers (and institutions) with an overlay of Average publication years weighted by citations. (Map illustrating authors with at least four publications in the area of research), with overlay of associated countries and thematic areas. **(C)** Term map based on co-occurrence of text in both the title and abstract fields using 212 publications based on *Sceletium* research. **(D)** Term map of research related to *Sceletium* with an overlay of the trend in citations over time. Data extracted from Web of Science (n = 212) and visualised with VOSviewer.

Several different research themes appear to be of superior relevance (as indicated by citation trends) in *Sceletium* literature. There were 349 terms that occurred three or more times in the 296 articles (**Figure 6** block D); these were separated into 13 thematic clusters identified through the VOSviewer (**Figure 6** block C). An analysis of the citations from 1956 to 2023 suggests that research associated with neurological disorders (ageing, depression, and anxiety) received significantly more citations per article (110, 55, and 41 average citations, respectively). This can be seen by the red-coloured bubbles (**Figure 6** block D). The neurological topics of ageing, anxiety, and depression had an average of 110, 75, and 40.7 citations each, respectively. Other topics that were relatively highly cited were terms associated with the chemical synthesis of mesembrine alkaloids. These terms, C-H-amination, Claisen rearrangement, cobalt catalysis, and enantiospecific synthesis, had average citation values of 45, 40, 31, and 58, respectively.

### Drivers of research

The drivers of research in terms of authors came from 17 authors who had contributed findings associated with *Sceletium* and mesembrine (**Figure 6B**). These authors were selected on the basis of contributing four or more publications from 1956 to 2023. Amongst the authors, three networks can be observed. The network from South Africa is the greatest contributor in terms of publications, with the leading author contributing 15 papers on the topic. This may be due to their location which allows ease of access to wild-growing plant materials and established working laboratory methods, where plant material is sourced through permits for collection that is not destructive. The network from Egypt contributed 13 documents on the topic. Presently, within South Africa, the Tshwane University of Technology (averaging 11 citations per year) and Stellenbosch University (averaging on 10 citations per year) are the major contributors to research and have contributed the most publications with 18 and 10 publications, respectively.

In terms of highly cited papers, with regard to chemistry from 1967 to 2000, the focus was largely on the isolation and characterisation of alkaloids from *Sceletium* species. Post-2000, the focus shifted to more chemical assays in an effort to develop quality control tools for the medicinally important plant, *S. tortuosum*, that was gaining pharmacological traction in literature as a phytomedicine used for anxiety and depression and as a mental stimulant. VOSviewer maps and an analysis of the literature indicate that the field may be shifting toward a greater focus on the toxicological and pharmacodynamics aspects of these plants (**Figure 6C**). Gaps in the field of pharmacology in the field were identified as clinical trials and bioavailability studies.

We observed 29 countries/territories with the highest contributing countries being South Africa (50 documents), China (29 documents), and the USA (23 documents) where scientific investigations in *Sceletium* have been conducted. All three are associated in a network and, as such, have exchanged techniques and gained access to analytical tools for more advanced chemical and pharmacological analysis. South Africa is suspected to be the greatest contributor to current research efforts, and this may be due to South African researchers having easier access to plant materials that grow in remote locations in the country and their compliance with Biodiversity laws that govern the issue of collection permits and bioprospecting activities linked to indigenous and endemic plant species in the country. Outside of this collaboration network, India has contributed 14 documents without collaboration with South Africa and these documents mainly cover topics related to the synthesis of mesembrine and joubertiamine alkaloids from *Sceletium* and one paper on the quality control of medicinal plants (Kumar and Sharma, 2018).

### Analytical chemistry

The bibliometric analysis, performed in VOSviewer, identified alkaloid chemistry and analytical techniques have been a major area of interest for investigations linked to *Sceletium*. This is seen as the most dominant cluster in terms of publications, indicated by a red cluster. The most commonly used mesembrine alkaloid biomarkers in quality control and analysis are mesembrine (**Figure 2A**), mesembrenone (**Figure 2B**), mesembranol (**Figure 6**, compound 2), and mesembrenol (**Figure 6**, compound 3). Some alkaloid classes which have been underrepresented in the literature that may hold medicinal activity but have not been tested yet are joubertiamine (**Figure 6**, compound 5), sceletium alkaloid A_4_ (**Figure 6**, compound 6), and the tortuosamine alkaloid classes of compounds (**Figure 6**, compound 8), which are also found in *Sceletium* species. The purple cluster represents experimentation related to the isolation and identification of compounds by various analytical means (**Figure 6C**). The body of work is quite substantial and has had a wide range of analytical techniques applied to the phytochemical characterisation of *Sceletium* species and related commercial products (**Table 2**). The majority of analytical methods used on *S. tortuosum* have targeted the detection of two alkaloids, mesembrine and mesembrenone (**Table 2**).

**Table 2:** The analytical techniques used on *Sceletium* species of medicinal importance, with their extraction method, sample preparation, and detectors (LE, liquid extraction).

Species	Plant parts	Biological matrices and plant specimens	Extraction method	Extract	Chemicals detected	Analytical techniques	Detectors	Reference
***Sceletium crassicaule*** (Haw.) L. Bolus (Syn. *Mesembryanthemum crassicaule* L. Bolus)	Aerial	Plant material	LE^1^	Methanol, dichloromethane	Sceletium alkaloid A4	ESI-MS, MS-MS, HPLC-UV	MS, UV	(Patnala and Kanfer, 2015)
	Aerial	Plant material	Acid/base extraction	Methanol, ammonia (25% w/w), sulphuric acid (98% w/w), and dichloromethane	Mesembrenol Mesembranol Mesembrenone Mesembrine	UPLC-PDA	PDA	(Shikanga et al., 2013)
***Sceletium expansum*** (L.) L. Bolus (Syn. *Mesembryanthemum expansum* L. Bolus)	Aerial	Plant material	LE	Ethanol	Alkaloid hordenine Joubertiamine dihydrojoubertiamine dehydrojoubertiamine	^1^H-NMR,^13^C-NMR, UV	UV	(Arndt and Kruger, 1970)
***Sceletium strictum*** L. Bolus (Syn. *Mesembryanthemum strictum*L. Bolus)	Not reported	Not reported	Not reported	Not reported	4′-O-Demethylmesembrenone mesembrenone Channaine	IR, ^1^H-NMR, MS	MS, IR	(Abou-Donia et al., 1978)
	Root, stem, and leaf	Plant material	Soxhlet extraction	Ethanol	Sceletenone Sceletium alkaloid A4, N-formyltortuosamine, 4′-O-demethylmesembrenone, Δ^7^-mesembrenone	^1^H-NMR, 13C-NMR, GLC-MS	MS	(Jeffs et al., 1974b)
	Root, stem, and leaf	Plant material	Soxhlet extraction	Ethanol	4′-O-Demethylmesembranol 4′-O-Demethylmesembrenol Mesembrenol O-Acetylmesembrenol	^1^H-NMR, ^13^C-NMR, GLPC-MS	MS	(Jeffs et al., 1970)
***Sceletium subvelutium*** L. Bolus (Syn. *Mesembryanthemum varians*)	Root, stem, and leaf	Plant material	LE	Methanol	(−)-3′-Methoxy-O-methyljoubertiamine (4R)-(−)-O-Methyljoubertiamine Joubertiamine, dihydrojoubertiamine O-Methyldihydrojoubertiamine	PTLC, MS, ^1^H-NMR, IR, UV	IR, UV, MS	(Nieuwenhuis et al., 1981)
***Sceletium tortuosum*** (L.) N.E. Br. (Syn. *Mesembryanthemum tortuosum* L.; *Kanna*)	Not applicable	Isolated compounds	Not applicable	Methanol	Mesembrenol Mesembrenol Mesembrenone Mesembrine	UHPLC-MS-qToF	MS-qToF	(Maphanga et al., 2022)
	Aerial and stems	Kanna powder, foliage, stems, and shredded material	LE	Methanol	Hordenine Mesembrenone Mesembrine Mesembrenol Mesembrinol	DART-HRMS	HRMS	(Appley et al., 2022)
	Aerial	Plant material (tissue cultured)	LE	Methanol	N-Demethylmesembrenol 4′-O-Demethylmesembrenol Joubertiamine 4′-O-Demethylmesembrenone Δ^4^-Mesembrenone Mesembrenol Mesembrine Mesembrenol Δ^7^-Mesembrenone	UHPLC-MS-qToF	QToF-MS	(Makunga et al., 2022)
	Aerial	Zembrin	LE	Water and ethanol	Mesembranol Mesembrenol Mesembrenone Mesembrine	UPLC-MS-PDA	MS, PDA	(Gericke et al., 2022)
	Aerial	Plant material	LE	Methanol	Sceletorine A Sceletorine B	^1^H-NMR ^13^C-NMR, UV-vis, IR, QSTAR ToF, HPLC-UV	UV-diode	(Yin et al., 2019)
	Aerial	Plant material	LE	Methanol	Mesembrenol Mesembrenone Mesembranol N-Demethyl-N-formyl Mesembrenone, Mesembrine Sceletium alkaloid A4 Δ^7^-Mesembrenone	^1^H-NMR, UPLC-MS	MS	(Zhao et al., 2018)
	Aerial	Plant material	LE	Methanol	Dihydrojoubertiamine, mesembrenone-M (O-demethyl-) Mesembrenone-M (O-demethyl-dihydro-) Mesembrenone-M (N-demethyl-dihydro-) Mesembrenone Mesembrine Mesembrine-M (dihydro-) Mesembrine-M (N-demethyl-) Mesembrine-M (O-demethyl-)	Leaf spray-MS	MS	(Freund et al., 2018)
	Aerial	Plant material	LE	Acetonitrile	Mesembranol Mesembrenone Mesembrine	UPLC-MS	MS	(Sandasi et al., 2018)
	Aerial	Plant material	Acid/base extraction	Methanol	Chanaine	HPLC-MS-PDA ^1^H-NMR	MS, PDA	(Veale et al., 2018)
	Aerial	Plant material	LE	Methanol	Mesembrenone mesembrine	UHPLC‐QToF‐MS	QToF, MS	(Manda et al., 2017)
	Aerial	Kanna powder	N/A^2^	N/A	4-O-Demetheylmesembranol 4-O-Desmethylmesembrenone, 4-O-desmethylmesembrenol Dihydrojoubertiamine Joubertiamine Mesembrane Mesembranol Mesembrenone Mesembrine O-Methyldehydrojoubertiamine O-Methyljoubertiamine Sceletenone, dehydrojoubertiamine	DART-HRToF-MS	HRToF, MS	(Lesiak et al., 2016)
	Aerial	Plant material	LE	Methanol, dichloromethane	Δ^7^-Mesembrenone Epimesembranol Mesembranol Mesembrenol Mesembrenone Mesembrine Sceletium alkaloid A4	ESI-MS, MS-MS, HPLC-UV	MS, UV	(Patnala and Kanfer, 2015)
	Aerial	Plant material	Soxhlet extraction	n-Pentane:n-hexane (1:1 v/v)	Bis-Demethyl-dihydromesembrine Mesembranol Mesembrenone Mesembrine N-Demethyl-dihydromesembrine N-Demethylmesembrenone, N-demethyldihydromesembrenone O-Demethyl-dihydromesembrine	^1^H-NMR, GC-MS, LC-(HR)-MS^n^	MS, (HR)-MS^n^	(Meyer et al., 2015)
	Aerial	Plant material	Acid/base extraction	Methanol, ammonia (25% w/w), sulphuric acid (98% w/w), and dichloromethane	Mesembranol Mesembrenol Mesembrenone Mesembrine	UPLC-PDA	PDA	(Shikanga et al., 2013)
	Aerial	Plant material, Kanna powder	LLE	Methanol, dichloromethane, ammonia (25% w/w solution), and sulfuric acid (H_2_SO_4_; 98.08% w/w)	Mesembranol Mesembrenol Mesembrenone Mesembrine	TLC, HPLC, GC–MS and ^1^H-NMR ^13^C-NMR (1 and 2D)	MS	(Shikanga et al., 2013)
	Aerial	Plant material, Kanna powder	Acid/base extraction	Methanol	Mesembranol Mesembrenol Mesembrenone Mesembrine	TLC, RP‐UHPLC-PDA, GC-MS ^1^H-NMR ^13^C-NMR (1 and 2D)	PDA, MS	(Shikanga et al., 2012e)
	Aerial	Plant material, Kanna powder	Acid/base extraction	Methanol	Mesembranol Mesembrenol Mesembrenone Mesembrine	HPTLC, GC-MS	MS	(Shikanga et al., 2012b)
	Aerial	Plant material, Kanna powder	LE	Methanol	Δ^7^-4′-O-Demethylmesembranol 4′-O-Demethylmesembrine 4′-O-Demethylmesmbranol Mesembranol Mesembrine	NACE-MS	MS	(Roscher et al., 2012)
	Aerial	Plant material	Acid/base extraction	Dichloromethane	Mesembranol Mesembrenol Mesembrenone Mesembrine	HSCCC, CC/PTLC, ^1^H-NMR GC-MS	MS	(Shikanga et al., 2011)
	Aerial	Plant material	LE	Methanol	Epimesembranol Mesembranol Mesembrenone Mesembrine Δ^7^-Mesembrenone	HPLC-UV, HPLC-PDA	PDA, UV	(Patnala and Kanfer, 2010)
	Aerial	Plant material	LE	Methanol	4′-O-Demethylmesembrenol Mesembrine Δ^7^-Mesembrenone	LC–UV-MS, HPLC-PDA	MS, UV, PDA	(Patnala and Kanfer, 2009)
	Not reported	*Sceletium* tablets	LE	Methanol	Epimesembranol Mesembranol Mesembrenol Δ^7^-Mesembrenone Mesembrenone Mesembrine	CZE-MS	MS	(Patnala and Kanfer, 2008)
	Root, stem, and leaf	Plant material	Soxhlet extraction	Ethanol	4′-O-Demethylmesembrenol Mesembrenone Mesembrine	GC-NPD-MS, TLC	NPD, MS	(Smith et al., 1998)
	Aerial	Plant material	Acid/base extraction	Chloroform	(+)-N-Acetyltortuosamine (+)-N-Formyltortuosamine 3′-Methoxy-4′-O-methyljoubertiaminol Joubetiamine Mesembrine Sceletium alkaloid A4 Tortuosamine	GLC,^1^H-NMR,IR	IR	(Jeffs et al., 1982)
	Root, stem, and leaf	Plant material	LE	Methanol	Sceletium A4 Unnamed alkaloid	GC-MS, TLC, ^1^H-NMR, IR	MS, IR	(Gross et al., 1979)
	Root, stem, and leaf	Plant material	Soxhlet extraction	Ethanol	4′-O-Demethylmesembrenone, sceletium alkaloid A4 Mesembrenone Mesembrine N-Formyltortuosamine Sceletenone Tortuosamine Δ^7^-Mesembrenone	^1^H-NMR, ^13^C-NMR, GLC-MS	MS	(Jeffs et al., 1974b)
	Root, stem, and leaf	Plant material	Soxhlet extraction	Ethanol	Sceletium alkaloid A4	^1^H-NMR, ^13^C-NMR, GLC-MS	MS	(Jeffs and Capps, 1979)
	Aerial	Plant material	LE	Ethanol	Dehydrojoubertiamine Dihydrojoubertiamine Hordenine Joubertiamine	^1^H-NMR, ^13^C-NMR, UV	UV	(Arndt and Kruger, 1970)

**Figure 7:**
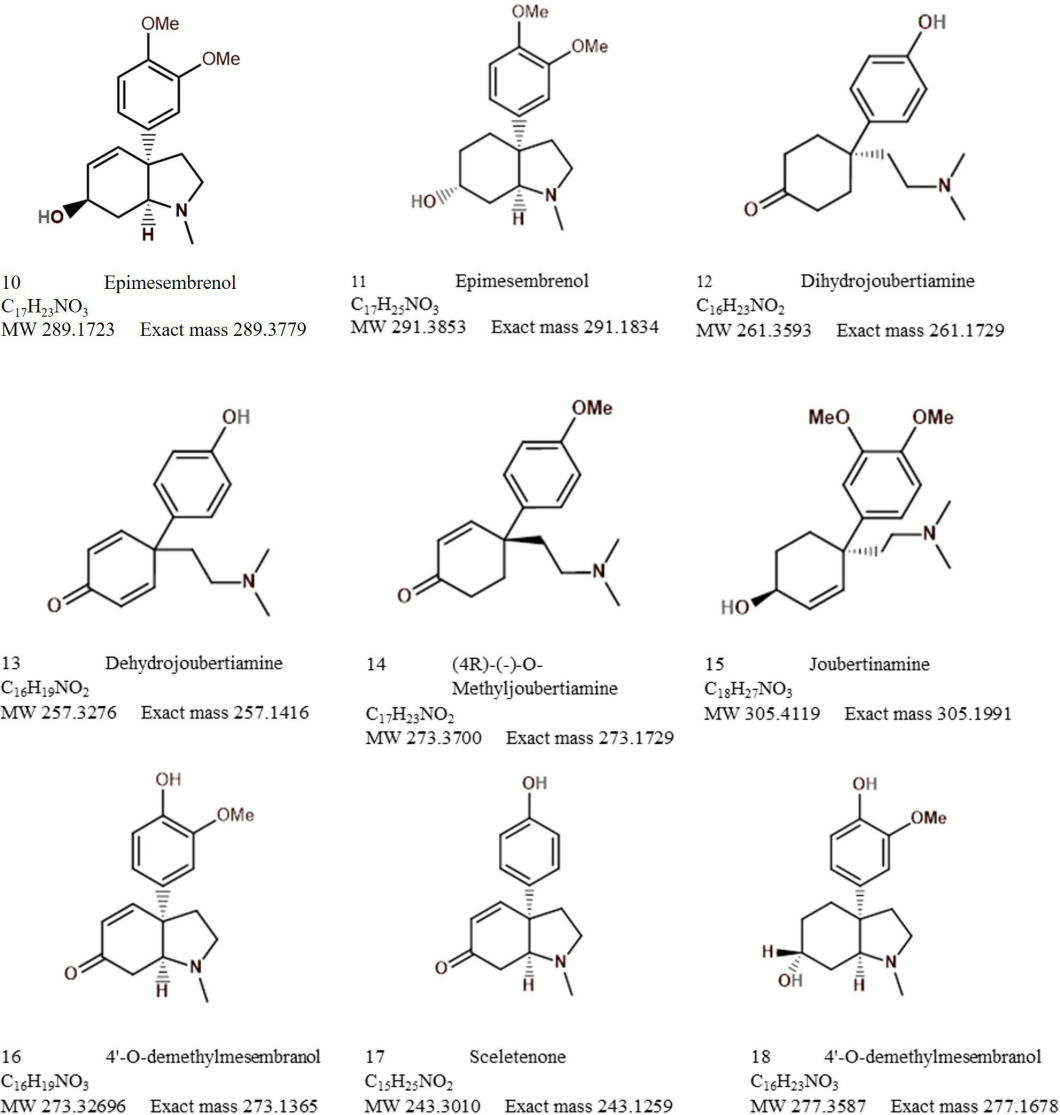
Mesembrine alkaloids from *Sceletium* of notable medicinal activity.

**Figure 8:**
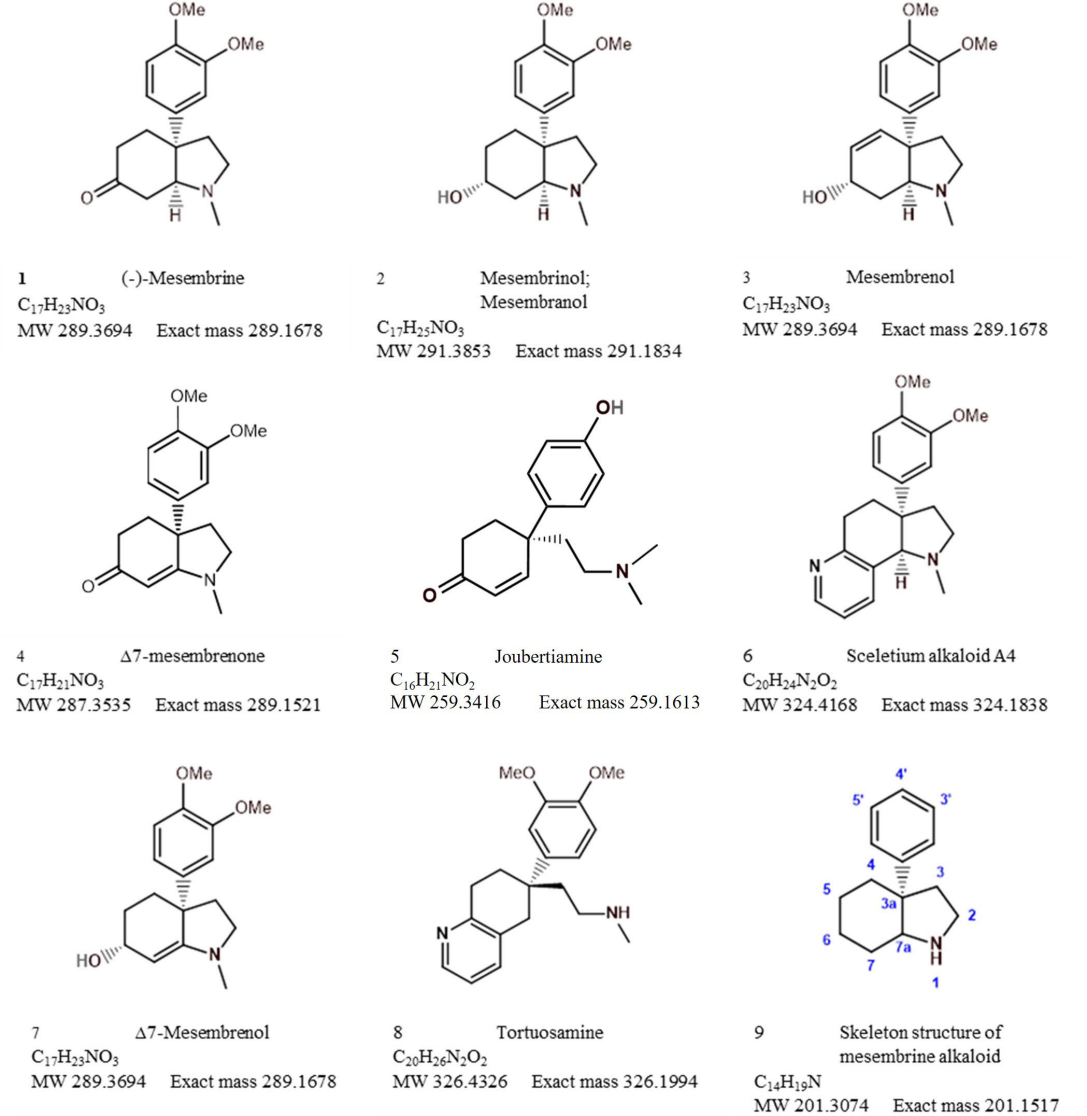
Other alkaloids found in *Sceletium* species.

**Figure 9:**
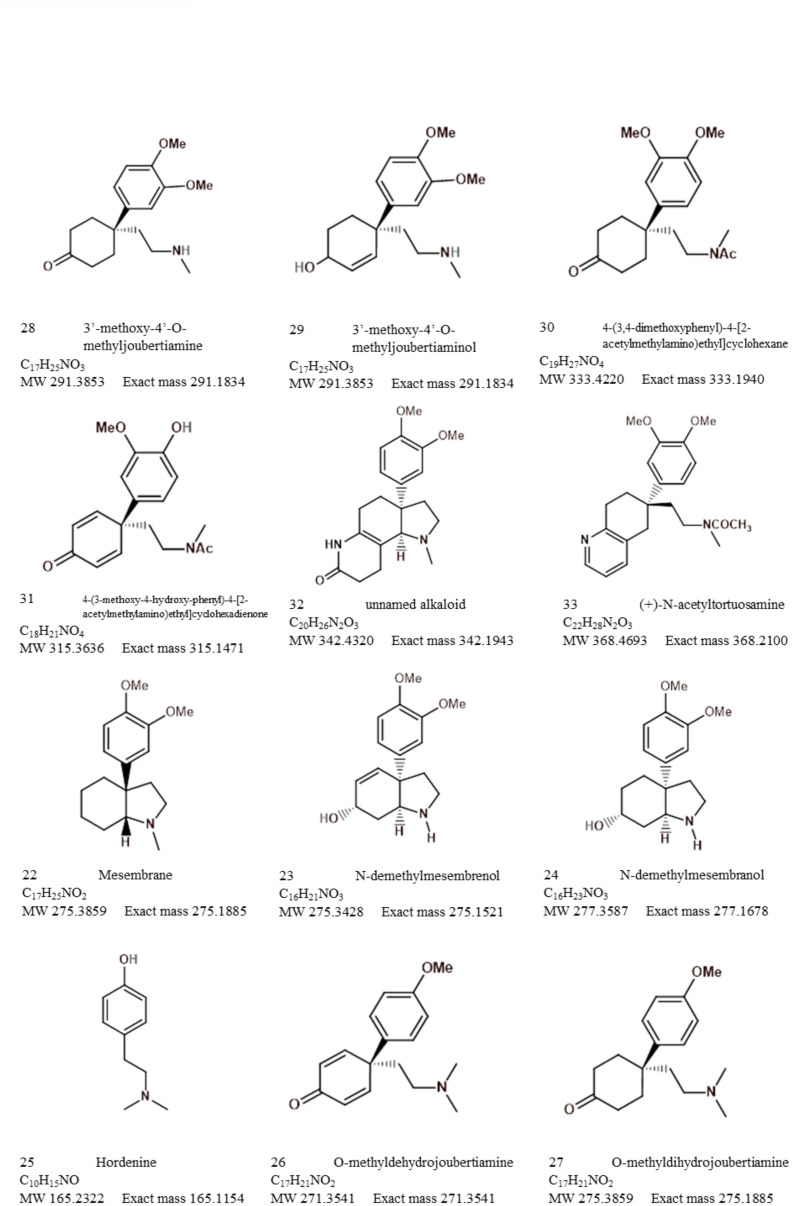
Other alkaloids found in *Sceletium* species (cont.).

**Figure 10:**
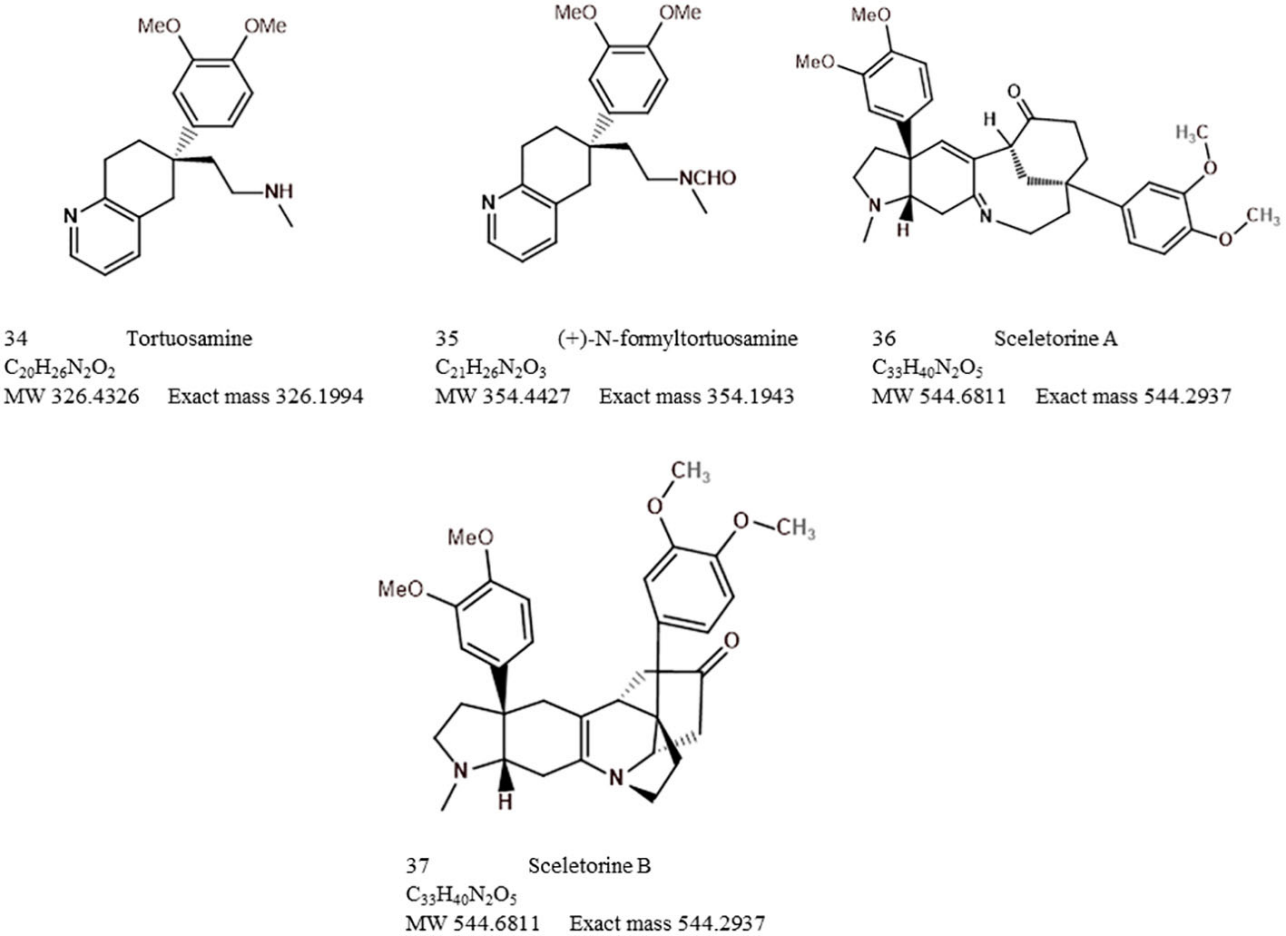
Other alkaloids found in *Sceletium* species (cont.).

Analytical chemistry in *Sceletium* has been of scientific interest since the 1970s. Many of the studies in the 1970s were mainly focused on compound isolation and structural elucidation (Jeffs et al., 1970, 1974b, 1974a; Arndt and Kruger, 1971; Abou-Donia et al., 1978; Nieuwenhuis et al., 1981) with a diversity of alkaloids as seen in **Figures 7**–**10**. In terms of *Sceletium*, however, the isolated structures did not necessarily enter into a drug discovery pipeline during the period of 1970 to 1998. The analysis of crude extracts using a variety of techniques, from thin layer chromatography, gas chromatography, and liquid chromatography, is more evident in the literature, and various laboratories have published several papers that focus on analysing mesembrine alkaloids. With changes in research foci in the natural products industry, where the study of complex plant mixtures using metabolomics in the 2000s till present has become an established field, smaller quantities of plant materials are being utilised than large amounts that were needed for isolation in the 1970s. Secondly, the focus has shifted to quality assessment of wild-harvested *Sceletium* species, as a means to compare wild populations to define chemotypes that occur naturally. Also, such application of metabolomics is explored for its potential contribution to the development of quality assurance protocols to ensure that *Sceletium*-based products are scientifically verified to contain the biomarker mesembrine alkaloids, which define their biochemical makeup (Masondo and Makunga, 2019). In a chronological format, using examples we highlight, the different analytical methods that have been used in *Sceletium* phytochemical studies are discussed below.

Jeffs et al. (1970) performed an ethanol extraction on *S. strictum* plant material. The crude alkaloid fraction was then analysed by chromatography. The GLPC allowed for the collection of 7 mg of an unidentified compound, 32 mg of mesembrenone, a 13-mg mixture of mesembrine–mesembrenone, 285 mg mesembrenol, 871 mg mesembrenol–mesembranol (at a ratio of 90:10 (w/w)), and 101 mg mesembrine. All of the structural identifications were possible and were achieved after nuclear magnetic resonance (NMR).

Sceletium A_4_ is one of the compounds that is now regarded as one of the biomarker compounds of *S. tortuosum*, and together with mesembrine, mesembrenone mesembranol, and mesembrenol (**Figure 2**), it can be used to differentiate wild-harvested chemotypes (Masondo and Makunga, 2019). The paper of Jeffs et al. (1971b) identified this particular compound, Sceletium A_4_ from *S. namaquense* (syn. *S. tortuosum*), for the first time. However, they did not report on any chromatographic techniques or quantitative data and information on structural characteristics from NMR data was solely presented.

Jeffs et al. (1974b) identified the alkaloids Sceletium alkaloid A_4_, tortuosamine, N-formyltortuosamine, and sceletenone isolated from *S. namaquense* (syn. *S. tortuosum*). Using X-ray crystallography, Abou-Donia et al. (1978) identified the structure channaine from *S. strictum*. However, the authors cautioned and suspected that channaine may have been an artefact from the condensation of two *N*-demethylmesembrenone molecules during the isolation process. With the exception of the recent study by Veale et al. (2018), discussed below in detail, no other reports have shown this unusual alkaloid to occur in *Sceletium* plants since then.

There is a clear lack of analytical isolation methods being used in studying the chemical constituents of *S. tortuosum* and its relatives from the 1980s till 1998. Renewed interest in these species is evident thereafter, with the work of Smith et al. (1998) that tested 21 species from nine genera of the Mesembryanthemaceae, for the distribution of mesembrine alkaloids. As compared with previous studies, significantly less plant material was used per extraction with methods staying relatively the same. The analytical techniques had advanced quite notably with the last research on *Sceletium* and its alkaloids that had been performed 16 years prior. Many investigations after 1998 that use smaller amounts of the plant sample as techniques that are in routine use for metabolite profiling are much more robust. Only semiquantitative data could be generated for this study due to no standards being readily available at that particular time. Out of the species tested, the only species with comparable mesembrine alkaloid levels to that of the *S. tortuosum* was *Aptenia cordifolia*. The relative levels of mesembrine were not reported on nor were *m/z* data for the other species presented.

Methods that allow for high-throughput detection of mesembrine alkaloids are thus sought after for industrial applications. Such methods also need to be less labour intensive and not necessarily require a high level of technical know-how for them to be placed in routine use, more especially to use them as a quality assurance measure and for the standardisation of manufactured products derived from *Sceletium*. As an example, Patnala and Kanfer (2008) investigated the analytical technique of capillary electrophoresis (CE), and this is a technique where electrophoretic mobilities under the influence of an applied electric field enable the separation of charged components. This analytical technique allows for the rapid and efficient separation of compounds leading to rapid analysis (Li, 1992). The technique is favourable due to its high efficacy and efficiency, as well as wide application for both scientific laboratories and industrial manufacturers, plus it requires low running costs during experimentation. Before this study, there was a paucity of reports on commercialised products of *S. tortuosum* despite an industry that had become established in South Africa. The study found the average content of mesembrine per tablet to be 164.30 μg per 12-mg dose of a tablet. Sensitivity and reproducibility were also an important consideration, and the authors confirmed that their protocol was both sensitive and reproducible. However, the exact species of *Sceletium* is not reported on in this study, which hinders the reproducibility of this work as one can merely assume that the focus was on the commercialised plant, *S. tortuosum*. It should be noted that there were some encountered difficulties during experimentation as the method could not conclusively distinguish between compounds with similar *m/z* values (diastereomers at *m/z* 292). Correct taxonomic identities for *Sceletium* species need to be accurate as these plants are difficult to distinguish from their anatomical structures and chemotaxonomic markers have not always played a significant role in delineating sister species from each other (Patnala and Kanfer, 2013).

The analytical tools chosen by Patnala and Kanfer (2009) were HPLC-PDA and LC-MS with a UV detector for qualitative and quantitative analyses of fermented and extracted materials. Fermentation resulted in the transformation of mesembrine. This subsequently led to lower levels being detected, which confounded evidence presented by Smith et al. (1998). Although precision and sensitivity were reported on, the authors did not provide detailed information relating to instrument conditions, making this study rather difficult to reproduce in other laboratory environments. In another study, Patnala and Kanfer (2010) developed and validated an HPLC method for the analysis of *Sceletium* plant material but the exact species used for this investigation was not indicated and one assumes that *S. tortuosum* was the target species. The method showed repeatable, precise, and appropriate resolution of alkaloids for quality control of mesembrine-type alkaloids. Prior to this study, poor validation data had been presented on any analytical techniques described for *Sceletium.* The analytical tools used were an HPLC system connected to a UV and PDA detector. Further structural data were supported by NMR spectra. The lack of standards resulted in several alkaloids assayed *via* chromatography whose identities were unknown to the researchers as no published data on these metabolites was available. Unfortunately, this study did not report on what species of *Sceletium* they had extracted the phytochemicals from, which is an issue in the reproducibility of this study. To iterate, this is highly problematic when some of the *Sceletium* species are difficult to distinguish from each other as they are similar in their appearance and their taxonomy is rather ambiguous (Patnala and Kanfer, 2013). The provenance of the plant samples can alter their phytochemical composition as many different chemotype configurations may exist in wild-collected populations, exhibiting both intra- and inter-specific variability (Shikanga et al., 2012c).

Due to the complex mixture of structurally similar alkaloids, the development of appropriate analytical techniques for chemotaxonomic assessment has proven to be quite a challenge. Further compounding this issue is that, in some species, for example, *S. emarcidum*, the distribution of alkaloids falls below the limit of quantification by the analytical tool (Patnala and Kanfer, 2013). To reduce these challenges, the introduction of reference compounds for all the alkaloids of interest may allow for better specificity during the fingerprinting process, assisting with the assay of plants with stronger precision and accuracy.

Shikanga et al. (2011) employed a high-speed countercurrent chromatography (HSCCC) method to rapidly isolate alkaloids from *S. tortuosum* in high yields. The quantity and purity obtained by HSCCC were higher in all alkaloids as compared with CC/PTLC also performed in this study. The method was efficient and cost effective, requiring relatively smaller amounts of plant material in isolating mesembrine (482.4 mg), mesembrenone (545.2 mg), mesembrenol (300.0 mg), and mesembranol (47.8 mg).

Chemotypic variation observed in *Sceletium* (Roscher et al., 2012; Shikanga et al., 2012c; Zhao et al., 2018) may be due to the ability of plants to exhibit phenotypic plasticity to cope with their environments and climates (Nicotra et al., 2010). Phenotypic plasticity enables plants with a standard genome to adapt their phenotype in response to environmental pressures assisting with survival (Nicotra et al., 2010). This phenotypic plasticity is often correlated with metabolomic differences in the plants in response to their environments; an example of this was observed in *Hippophae rhamnoides* (Kortesniemi et al., 2017). The study of chemical variation linked to plant-environment effects can thus easily be achieved using phytochemical analytical techniques.

The field of plant metabolomics is making major contributions to our understanding of plant biochemistry and metabolism as a metabolomics workflow can facilitate a comprehensive compilation of metabolites within a particular cell, tissue, or organ, but large-scale experiments are notoriously difficult to interpret. In such instances, the complexity of these data sets is enormous and they cannot easily be processed with classical statistics (Van der Kooy et al., 2008), consequently principal component analysis (PCA), and partial least squares (PLS) analysis have been employed. These types of multivariate statistical applications reduce the dimensionality of the data enabling better pattern recognition that can be correlated with the analysed samples.

Shikanga et al. (2012c) further developed a method for the rapid and simple identification of alkaloids in *S. tortuosum* raw and wild-harvested materials. The intended purpose of this study was to develop an analytical technique for the routine analysis of psychoactive alkaloids in *S. tortuosum* these products. The analytical tool used was a high-performance thin-layer chromatography (HPTLC) densitometric method as this is a superior and more sophisticated form of TLC that is fast and robust for quality testing of botanical materials. One of the advantages of using this method is the automation of the different steps that would mainly be performed by hand with a normal TLC. This makes this method more powerful for metabolite fingerprinting, increasing its resolution and enabling quantitative measurement of phytochemicals.

The first study to use the analytical technique of non-aqueous capillary electrophoresis coupled to mass spectrometry (NACE-MS) was aimed at analysing wild and commercial plant materials extracted from methanol as a solvent from *S. joubertii* (syn. *S. tortuosum*) (Roscher et al., 2012). Another point of interest in this study was to investigate the influence fermentation would have on alkaloid profiles. Wild (calyx, stems, and leaves) and commercial plant material was extracted using methanol as a solvent. Samples were also fermented, and their alkaloid profiles were obtained. The fermentation was performed by crushing the whole wild harvested plant, and this included the calyx, stems, and leaves as plant parts. After homogenisation of the material, it was then stored in an airtight transparent plastic container, left in the sun for 8 days, and vacuum dried later. All analyses were performed on capillary electrophoresis coupled to an Ion Trap 6330. The high selectivity of this method is evident by its ability to distinguish the diastereomers of 4′-O-demethylmesembranol (retention time of 12.3 min); since then, no other analytical techniques have been able to identify it (Smith et al., 1998; Patnala and Kanfer, 2008, 2010). No quantification data were presented in this paper. Furthermore, no comparison is made with a reference technique with the same samples. The technique proved effective in the relative quantification of alkaloids from wild and commercial samples of *Sceletium*. However, the authors do not present any evidence validating the method in terms of linearity, limits of detection, and repeatability. Nevertheless, this technique provided novel opportunities to study samples that potentially have diastereomers and isobaric structures.

Shikanga et al. (2013) performed a study using UPLC and hyperspectral imaging to distinguish between *S. tortuosum* and *S. crassicaule* as these species are difficult to distinguish from each other as they look almost identical, often leading to their misidentification. Hyperspectral imaging is proving a valuable tool in the authentication of herbal products, but it is heavily reliant on good statistical models to make predictions after test materials have been scanned. Its main advantage is that it circumvents an extraction step using organic solvents, making it time efficient, non-destructive, and friendly to the environment and users. This was the first study to investigate the chemical composition of *S. crassicaule.* The purpose of this study was to offer an additional robust tool to reduce the adulteration of *Sceletium* with species that may contain fewer alkaloids of interest and ultimately assist in the authentication of *Sceletium* material. The hyperspectral method combined with chemometrics was thus substantially more efficient in the chemotaxonomic classification of *S. tortuosum* and related species.

Patnala and Kanfer (2013) performed a chemotypic analysis of six species that were selected based on venation patterns as distinguishing morphological characteristics are often used in taxonomy to assign species identities. The plants were grouped into the ‘tortuosum’ (*S. tortuosum*, *S. expansum*, and *S. strictum*) or ‘emarcidum’ type (*S. emarcidum*, *S. exaltum*, and *S. rigidum*). The species of *S. varians* and *S. archeri* were not considered in this study; in fact, these species have largely been ignored in terms of their phytochemical profiles. The ‘tortuosum’-type plants, *S. tortuosum* and *S. expansum* were predominantly characterised by the presence of mesembrine, mesembrenone, mesembranol, and epimesembranol. *S. strictum* was found to contain measurable amounts of mesembrine, mesembrenone, and one of two epimers, 4′-O-demethyl-mesembrenone or 4′-O-demethyl-mesembrenol, but mesembranol and epimesembranol were in minute relative amounts. Interestingly, the ‘émarcidum’ types illustrated a complete absence of the mesembrine class of alkaloids traditionally associated with *Sceletium* such as mesembrine, Δ^7^mesembrenone, mesembrenone, and mesembranol. Instead, the émarcidum group had O-demethyl-mesembrenone and O-methyl-joubertiamine as the more prominent metabolites and out of the émarcidum’ types, *S. exaltum* showed an accumulation of mesembrine. It was concluded that the distribution of mesembrine-type alkaloids is not distributed across the genus and is limited to only a few species, highlighting the importance of quality control testing in the *Sceletium* genus.

It is hypothesised that the *Sceletium* genus may have recently diversified, with minimal time between speciation events (Klak et al., 2007). As a result, these species have had a very brief period of time to accumulate differences in their DNA and subsequently are very similar in morphology. Little information is currently available with respect to the chemical fingerprints of both *S. crassicaule* and *S. emarcidum*. Patnala and Kanfer (2015) analysed wild material of *S. crassicaule* and *S. emarcidum* using electrospray ionisation mass spectrometry (EI-MS) and LCMS to characterise the chemical fingerprints of specific *Sceletium* alkaloids as a tool for the qualitative identification of lesser investigated alkaloids with complex matrices such as Δ^7^ mesembrenone, Sceletium A_4_, and epimesembranol. Furthermore, the study assessed the potential of the analytical method as a tool in quality control of *Sceletium* commercial products as tablets derived from *S. tortuosum* were included in the analysis. Their technique successfully identified Δ^7^mesembrenone, mesembrenol, mesembrenone, Sceletium A_4_, mesembranol epimesembranol and mesembrine from several species of *Sceletium*.

The method identified Sceletium A_4_ from *S*. *crassicaule* material. Of interest, *S. emarcidum* did not have any of the reference compounds, which normally occur in *Sceletium* samples. The investigation did not report on any dominant structures that could be used for the chemotaxonomic classification of this species, as none of the peaks observed corresponded with the standard alkaloids found in *Sceletium* species.

Apart from plant misidentification and the choice of inferior chemotypes that express poor bioactivity, chemical and heavy metal adulterations as well as herbal adulterations of phytomedicines can lead to undesired cytotoxic effects upon human consumption. Metabolite profiling can thus be a complimentary tool to other techniques for the detection of adulterants in herbal medicines. To this end, Lesiak et al. (2016) performed analysis on *S. tortuosum* commercial material using direct analysis in real-time ionisation coupled with high-resolution time-of-flight mass spectrometry (DART-HRTOF-MS). The method was employed as an authentication tool to identify adulterated samples and found that some commercially available samples were indeed spiked with the banned herbal stimulant ephedrine. Commercial powder mixtures were conveniently analysed directly by dipping the closed end of a melting point capillary tube into the powder substance and then between the DART ion source and mass spectrometer inlet. The authors only quantified two of the detectable compounds, mesembranol ranging from 0.3% to 7.0% and mesembrine at 5.1%, but relative amounts are not available for any of the other compounds in the authors’ report. The method provided a rapid forensic diagnostic tool of commercial samples sold in the USA, highlighting illicit practices in the manufacture of *Sceletium*-derived products that are of regulatory concern.

Appley et al. (2022) was another analytical study concerned with the authentication of *Sceletium*-based products for the forensic analysis of products containing *Sceletium*, using robust protocol that detected hordenine and mesembrine-type alkaloids from *Sceletium*. Supporting the method used by Lesiak et al. (2016), the use of direct analysis in real time–high-resolution mass spectrometry (DART-HRMS) resulted in effective and rapid detection and quantification of hordenine and mesembrine-type alkaloids. Ephedrine is a concern in natural products as it is lethal when combined with caffeine or other over-the-counter drugs and could potentially be harmful when consumed with *Sceletium* products (Haller and Benowitz, 2000). An advantage of DART-HRTOF-MS is that sample preparation is not needed and thus not prone to solvent bias. Techniques such as LC-MS and GC-MS may not identify adulterants such as ephedrine due to preferential take-up of hordenine due to its polarity over the adulterant ephedrine as these are constitutional isomers of each other, which both occurred at a nominal *m/z* of 166. The authors emphasised that without the use of DART-HRTOF-MS, the compounds would not have been separated and the adulterant would have thus become more difficult to notice and identify.

The study of plant metabolomes has seen an unprecedented rise since the adoption of systems biology approaches in biological sciences and NMR metabolomics can be the preferred choice for this purpose (Verpoorte et al., 2007; Leiss et al., 2011). The advantage of using NMR for generating an overview of the plant metabolome lies in its vast applications, ranging from quality control of foods and botanicals to studies related to investigating the pharmacological activity of phytochemicals. A limitation of NMR spectroscopy is that, without the use of two-dimensional NMR, absolute quantitation is not possible (Verpoorte et al., 2007). The plants collected at different localities studied by Zhao et al. (2018) using NMR were growing under differing biogeographic environments of the Western Cape and Northern Cape. The study found that NMR chemometrics could be an effective tool to distinguish between populations of *Sceletium* and identify notable biomarkers in each population. *N*-Demethyl-*N*-formylmesembrenone, a biomarker that had not been identified in *Sceletium* before, characterised one of the population groups from the Western Cape. Furthermore, the production of alkaloids may be due to genetic composition rather than climatic conditions since plants in close proximity to each other produced variable amounts of alkaloids, suggesting that climate was not a contributing factor to diversity in chemical profiles. Freund et al. (2018) employed an analytical technique that had never been performed on these plants coined leaf spray mass spectrometry (leaf spray MS) (**Table 3**). Additionally, the setup included tandem mass spectra (MS/MS). This technique circumvents the separation of plant metabolites using chromatography and offers a direct MS injection without the need for sample preparation or extraction, with minimal technical adjustments to the ionisation source being required. An advantage of this tool is the absence of lengthy extraction protocols and solvent bias that may introduce artifacts or prove inefficient in the recovery of phytochemicals as these may not always be extracted. Leaf spray MS can directly analyse plant tissue providing rapid generation of qualitative and quantitative data albeit accurate quantification is more tricky with this approach. The method was successful in analysing intact plant material reducing the amount of processing needed. For leaf spray MS to have wider application for *in planta* analysis of metabolites, optimisations in terms of plant preparation, presence or absence of solvents, volume of solvents, voltage amplitude, and distance from the ion inlet may be necessary. Some other limitation of this analytical technique is its low dynamic range, resulting in the most abundant metabolites solely being identifiable. Some of the minor alkaloids or structurally similar alkaloids that require stronger resolving power require greater and more sophisticated technical expertise for their detection. Despite this, the analytical method proved to be a powerful tool that eliminated chromatography for the identification of the main phytochemicals from *S. tortuosum.*

**Table 3:** Methods used for fermentation and results obtained from previous research (↑ =increase, ↓ =decrease).

Extraction method	Fermentation procedure	Analytical technique	Conclusion	Reference
Classical acid–base extraction	Plants crushed with soil, placed in a plastic bag, and sealed in an airtight plastic bag.	GC-MS	↓ 4′-O-Demethylmesembrenol ↓mesembrine ↑mesembrenone	(Smith et al., 1998)
Methanol	Aerial plant parts crushed and placed in plastic bag exposed to sun for 10 days.	HPLC-MS (validated)	↓Mesembrine ↑ Δ^7^-Mesembrenone	(Patnala and Kanfer, 2009)
Methanol	Plant material stored in airtight plastic container for 8 days.	NACE-MS	Alkaloid profile remained unchanged, no quantitative data reported.	(Roscher et al., 2012)
Methanol	Aerial plant parts gently bruised and placed in an incubator (40°C) for 7 days	UPLC-MS (validated)	↑ Mesembrine ↓Mesembrenone	(Chen and Viljoen, 2019)

Sandasi et al. (2018) performed a study to assess the quality of herbal tea blends using hyperspectral imaging and UPLC-MS in various *Sceletium* tea blends. Five batches of herbal tea that were claimed by the manufacturers to contain a *S. tortuosum* and *Cyclopia genistoides* (commonly known as honeybush) mixture were obtained pulverised and subjected to hyperspectral imaging without any further processing. For UPLC-MS analysis, the tea blends were prepared by adding boiling water (237 ml) to 1.5 g of plant tissue. For the hyperspectral imaging, the same instrument settings were used as in Shikanga et al. (2013) and applied as a rapid and non-destructive method for the quality control of the tea blends. Using a PLS-DA model, the procedure had a 95.8% predictive ability providing high degrees of sensitivity with a stronger metabolite feature selection. Quantitatively, *C. genistoides* was found to be in higher amounts across the samples (>97%), whereas *S. tortuosum* was found in lower quantities (<3%). For this study, the UPLC-MS conditions were optimised for *C. genistoides* but these conditions were not necessarily optimal for *S. tortuosum*. A major limitation of this particular study was that the UPLC-MS procedure alone could thus not conclusively efficiently distinguish the plant components of the herbal mixtures that contained *S. tortuosum* along with another species. Combining hyperspectral imaging with chemometrics proved a more powerful and reproducible tool for the quality control of herbal tea blends containing *Sceletium* and honeybush.

It is not clear why there are so few reports on the isolation and characterisation of channaine; however, it is likely that analytical methods being used by researchers are not necessarily optimised for the detection of this unusual alkaloid channaine. We speculate that this compound may also be produced at minor levels during the lifetime of the plant, making it even more difficult to isolate. Veale et al. (2018) aimed to structurally elucidate the alkaloid, channaine from *S. tortuosum*. This was the second time that channaine was detected since its initial characterisation by Abou-Donia et al. (1978). This was the first full NMR analysis of channaine in the literature. Chemical structures were resolved using ^1^ H, ^13^C, COSY, HSQC, and HMBC NMR spectroscopy.

The search for novel chemicals from *Sceletium* species has found renewed interest. Recently, Yin et al. (2019) performed an extraction of *S. tortuosum* and isolated sceletorines A and B for the first time with these authors making suggestions on plausible biosynthetic pathways associated with sceletorine production. This kind of information is largely missing in terms of novel alkaloids that become periodically identified in *Sceletium* samples by different research groups. The isolated alkaloids were established to be precursors of the alkaloid channaine identified in previous studies (Abou-Donia et al., 1978; Veale et al., 2018). It was ruled out that these phytochemicals were artefacts as a result of processing due to their presence in fresh material. Sensitivity, reproducibility, and comparison with a reference method of these two new alkaloids were not presented in the paper.

Reddy et al. (2022) investigate the chemotypic variation across populations of *Sceletium* species. This is one of the few studies looking at the chemical composition of other species in the genus and the only one to report on the chemical composition of *S. rigidum* and *S. emarcidum*. The analytical technique of HPLC-MS-MS was employed, and data were processed using Feature-based Molecular Networking to annotate and investigate the chemical space in greater detail to identify minor and coeluting phytochemicals. The study put forward *in silico* results supporting that minor phytochemicals identified in *Sceletium* species may also be responsible for the therapeutic activities observed in the literature (Harvey et al., 2011; Krstenansky, 2017).

The main challenges associated with the purification and identification of *Sceletium* alkaloids are linked to irreversible adsorption to column packing materials, excessive tailing, and poor recovery as well as catalytic changes encountered with solid supports in various analytical systems (Yang and Ito, 2005). This can be overcome to some extent by high-speed counter-current chromatography (HSCCC) (Shikanga et al., 2011) and non-aqueous capillary electrophoresis coupled to mass spectrometry (NACE-MS) (Roscher et al., 2012).

Overall analytical techniques should be performed to assess sensitivity, reproducibility, and comparison with a reference method (i.e., GC-MS) (Shikanga et al., 2012c; Zhao et al., 2018). From the current state of analytical techniques used in the quality control of *Sceletium*, GC-MS, LC-MS, and HPLC-MS will continue to remain popular going forward (**Table 2**). However, for the effective identification of adulterants and contamination in samples, more advanced tools in tandem with different detectors need to be utilised. Two analytical techniques that stand out for the rapid analysis of samples are direct analysis in real-time ionisation coupled with high-resolution time-of-flight mass spectrometry (DART-HR-TOF-MS) (Lesiak et al., 2016) and leaf spray MS (Freund et al., 2018). These methods do not require the processing of material, and as such there is no solvent bias, loss of phytochemicals during extraction, or artifacts from extraction procedures. Nevertheless, the limitations of these methods are that the machines are not common, are expensive, and require specialised components that may prove to be more laborious to assemble. With these in mind, the application of NMR analysis coupled with chemometrics (Zhao et al., 2018) will also gain more popularity in quality control assurance practices. Non-destructive methods such as hyperspectral imaging may provide additional analytical power for use in commercial settings (Sandasi et al., 2016, 2018). Further advancements in analytical techniques will likely result in novel methods that may be used in the future.

**Figure 11:**
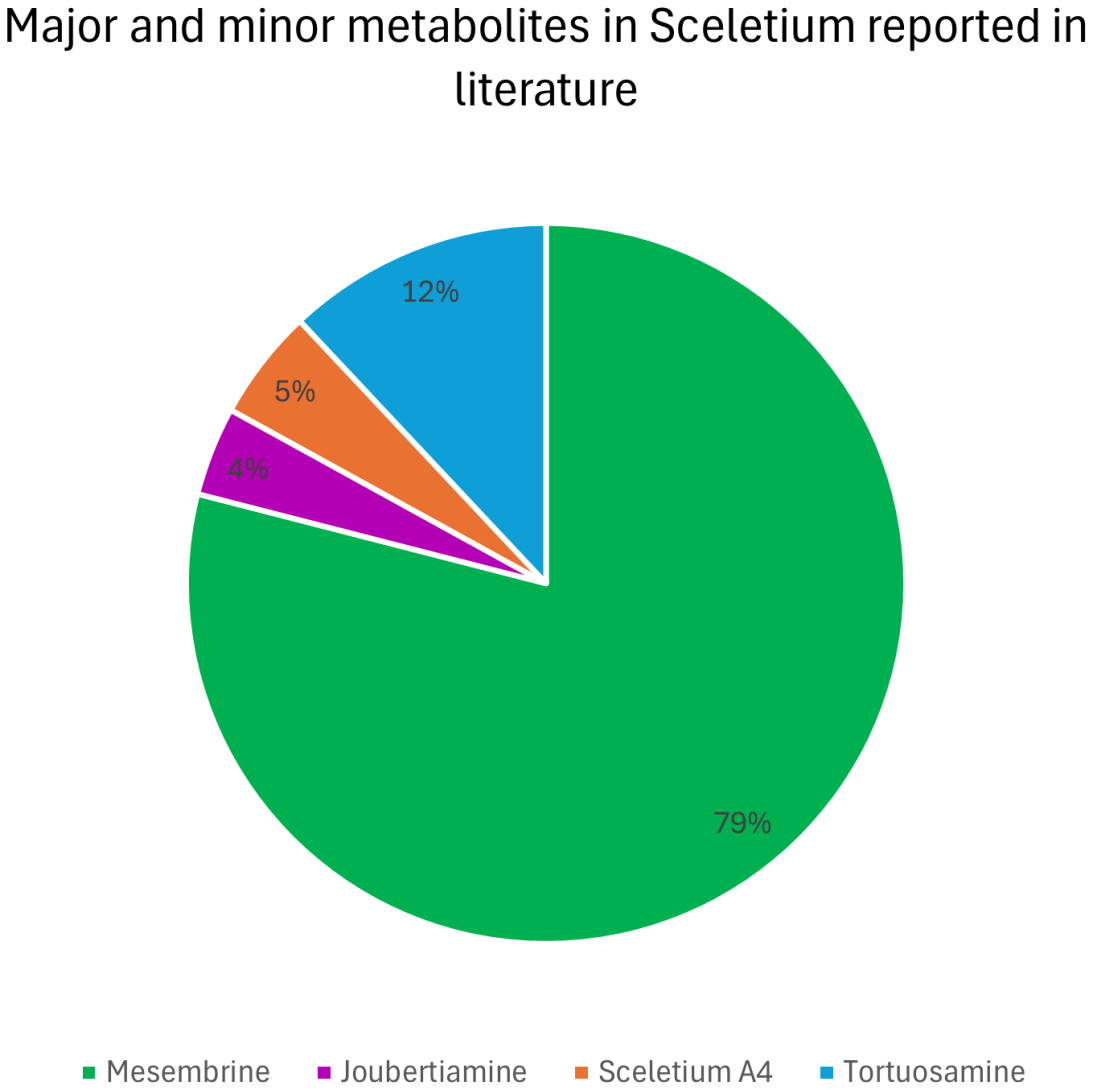
Alkaloid diversity reported based on scientific literature across analytical techniques used to study *Sceletium*.

The major and minor alkaloid classes amongst *Sceletium* species have been shown as a pie chart diagram (**Figure 11**). This diagram represents what past chemical analysis studies have reported to be detected in *Sceletium*. It should be noted that this is just representative of what the respective authors searched for (refer to **Table 2**) and not of the true alkaloidal distribution *in planta* in *Sceletium.* The distribution of major and minor alkaloid classes in different plants of *Sceletium* has been shown to be highly variable, and at times, plants from the same population may have differing amounts of a particular alkaloid; this has been hypothesised to be resultant from chemical plasticity that may be associated with environmental and genetic responses (Reddy et al., 2022).

### Fermentation of *Sceletium*

The fermentation of *Sceletium* and the effect on the medicinally important alkaloids have been of interest due to the traditional preparation and the anecdotal reports of the plant becoming more euphorically potent when fermented (Smith et al., 1996). Although the traditional method of using an animal skin bag is no longer used nowadays, fermentation is thought to enhance the levels of these alkaloids and reduce oxalates, which in turn increases the mood-elevating activity of *Sceletium* (Smith et al., 1996). There seems to be some incongruency in reports related to the effects of fermentation, which further highlights work that needs to be done to better understand the metabolic pathways of these alkaloids and at which steps to manipulate levels.

Many analyses have investigated the effect of fermentation on the alkaloid profile in *S. tortuosum* (**Table 3**). Smith et al. (1998) used the plastic bag fermentation followed by GC-MS analysis. Their findings indicated that the mesembrine alkaloid composition was comparable with that of the oven-dried (80°C) samples. However, in the fermented sample, there was a significant increase in mesembrenone levels whereas levels of 4′-*O*-demethylmesembrenol and mesembrine decreased. Patnala and Kanfer (2009) further investigated the role of fermentation in alkaloid composition in *Sceletium* using HPLC-MS, where levels of mesembrine decreased [from 1.33% to 0.05% (w/w)], with suspected transformation into Δ^7^mesembrenone. Roscher et al. (2012) also investigated the change in alkaloid composition as a result of sample fermentation. No qualitative data were presented in this study; instead, the authors report that no overall change in the alkaloid concentrations as a result of fermentation were detected. The studies conducted up until this point were suggestive that the traditional processing of the plant material through fermentation did not affect the overall potency of the material as there was a decrease or no change in mesembrine levels (Smith et al., 1998; Patnala and Kanfer, 2009; Roscher et al., 2012). However, the most recent study by Chen and Viljoen (2019) reported that the total alkaloid content increased as a result of fermentation. They reported that while mesembrine levels increased (from below 1.6 μg/mL to 7.40 μg/mL–20.8 μg/mL), there was only a marginal increase in mesembrenol and mesembranol content and a significant decrease in mesembrenone content. This study supports the traditional preparation of *Sceletium* plant material to increase the mood-elevating effects of *S. tortuosum.* It may be worthwhile to investigate if phytochemical formation or breakdown is dependent on pH in these analyses and that future analyses should control for this. Previous studies have not indicated the pH of their extracts or used them in their analyses.

Comparing the fermentation studies available in the literature thus far, it is clear that there is no conclusive evidence that fermentation results in a consistent change in the alkaloid profile. More investigation is needed to understand how fermentation affects alkaloids in *Sceletium* and what the best method of fermentation is that most accurately represents traditional ethnobotanical preparation. A better understanding of the biosynthetic pathway(s) could assist in understanding how fermentation influences the metabolite profile. Currently, the biosynthetic pathway of mesembrine-type alkaloids as suggested by Jeffs et al. (1971a) proposed that the perhydroindole portion of mesembrine comes from tyrosine and the aromatic group is derived from phenylalanine. The cinnamic acid derivatives are produced from phenylalanine, but the 3′-aryl oxygen substituent is proposed to be introduced in later steps involving a biosynthetic reaction with sceletenone, mesembrenone, and 4′O-demethylmesembrenone (Jeffs et al., 1978). Since studies in 1971 and 1978, the biosynthetic pathway has not been revised and we thus suggest this become a future avenue of investigation to better understand how fermentation affects the alkaloid profile, pinpointing not only biochemical changes that occur but also the key enzymes and genetic regulatory steps that may control this metabolism.

### Present-day ethnobotanical use

Although there is limited current ethnobotanical information on the prevalence of use of the *Sceletium* genus in modern times, the work by Philander (2011) focusing on a group of Rastafarian herbalists clearly points out to the importance of *S. tortuosum* as the main species that is collected as a phytomedicine to reduce depression and anxiety and in some cases, it is consumed together with *Cannabis sativa*, for spiritual purposes (Schell, 2014). With the increased public interest in biogenic drugs such as *Sceletium*, numerous companies have appeared online selling *Sceletium* in raw powdered form, tablets, teas, and snuffs. This increased popularity may well pose a significant conservation threat to the species if populations are collected from the wild. It appears that some of the present-day uses by indigenous communities of the plant are consistent with the historical uses, i.e., for euphoria and as a mood elevator (Smith et al., 1996; Gericke and Viljoen, 2008).

### Biological activities of *Sceletium* extracts and isolated alkaloids

There are limited reports that support the hunger and thirst suppression effects that are claimed of *Sceletium*. There are, however, several reports on the ‘mood elevation’ activity, particularly focussed on the potential of *Sceletium* to aid with anxiety and depression (**Table 4**). Recently, there have been more studies that are focused on the pharmacology of *Sceletium*, notably in areas linked to *in vivo* actions and clinical trials of tested extracts (**Table 4**). The body of pharmacology research on *Sceletium* is quite extensive, with the majority of the reported biological activity being interactions with the central nervous system (CNS) and related neurological pathways (antidepressant, anxiolytic, and psychoactive activity). The scope of the observed CNS activity is broad with observed anxiolytic and antidepressant activity demonstrated for extracts and isolated compounds of *Sceletium* (**Table 4**). Additionally, there are more *in vivo* behavioural inquiries on rats using a range of pharmaceutical tests (**Figure 6D**) exhibiting CNS-related activities ranging from suppressant (e.g., anxiolytic and sedative) to excitatory (e.g., antidepressant) activity. Although there is a shift to more *in vivo* studies, there is still much to be tested in terms of different chemotypes and to understand the pharmacokinetics of individual phytochemicals and potential synergism between phytochemicals, aside from mesembrine.

**Table 4:** The CNS-related activity of *Sceletium tortuosum* extracts and compounds (*Note that TRI is an extract of a *S. tortuosum* and *S. expansum* hybrid).

Effect	Compound/extract	Model/target	Positive control	Formulation/dosage	Result/mechanism/method	Reference
Alzheimer’s dementia	**Zembrin** extract	Neuropsychological tests: CNS Vital Signs and Hamilton depression rating scale (HAM-D) (targeted at PDE-4)	Placebo capsule, no herbal extract	*In vivo*—clinical trial 25 mg	Zembrin significantly improved cognitive flexibility (p < 0.022) and executive function (p < 0.032) as compared with placebo	(Chiu et al., 2014)
Analgesic	**Zembrin** extract	Rat pharmaco-EEG (Tele-Stereo-EEG)	0.9% NaCl (1 ml/kg), showed minor effects to delta and alpha2 power in frontal cortex	*In vivo* 1.0, 2.5, 5.0, and 10.0 mg/kg	Zembrin showed second strongest effects were reduction in both the delta and the theta signals	(Dimpfel et al., 2016)
	Full **alkaloid extract**, the **alkaloid enriched fraction**, and **mesembrine**	Hotplate assessment of analgesic activity	Morphine (5 mg/kg), increased hotplate latency response	*In vivo* 100, 20, and 20 mg/kg	Mesembrine showed an increased hotplate latency response.	(Loria et al., 2014)
Antidepressant	**Zembrin** extract	Flinders Sensitive Line (FSL) rat model in Forced swim assessment and open field test (OFT) of antidepressant activity	Escitalopram	*In vivo* 5, 10, 25, 50, or 100 mg/kg	Zembrin at doses of 25 mg/kg and 50 mg/kg significantly reduced immobility compared with saline controls—supporting dose-dependent antidepressant-like activity	(Gericke et al., 2022)
	**Chloroform extract** of *S. tortuosum*	Chick anxiety-depression model	Imipramine (10 mg/kg), exhibited no effects in anxiety phase but increased DVoc^3^ in early and late phase depression state.	*In vivo* 10, 20, 30, 50, 75, 100 mg/kg	At concentrations of 10 mg/kg-30 mg/kg extract showed no effect on depressive state. Concentration of 75 mg/kg-100 mg/kg exhibited no effect on depressive state.	(Carpenter et al., 2016)
	**Zembrin** extract	Rat pharmaco-EEG (Tele-Stereo-EEG)	Citicoline (46 mg/kg), rolipram (0.1 mg/kg	*in vivo* 1.0, 2.5, 5.0 and 10.0 mg/kg	Attenuation of alpha1 waves emerged during the highest dosage in all brain areas.	(Dimpfel et al., 2016)
	**Trimesemine** extract (TRI*)	Cell culture (SERT and VMAT-2: human astrocytes and murine hypothalamic neurons)	10 μM citalopram, significant downregulation of SERT and no effect on VMAT-2	*In vitro* 1.0, 0.1, 0.01, 0.001, and 0.0001 mg/m	1 mg/ml extract showed comparable activity with positive control (15 min, SERT), 1 mg/ml and 0.1 mg/ml extract showed comparable activity with citalopram (30 min, SERT). Significantly higher VMAT-2 expression noted at extract concentration of 1 ml/ml (15 min, VMAT-2)	(Coetzee et al., 2016)
	Full **alkaloid extract**, the **alkaloid enriched fraction**, and **mesembrine**	Forced swim assessment of antidepressant activity	Imipramine (15 mg/kg), decreased float time	*In vivo* 100, 20, and 20 mg/kg, respectively	Mean float time for alkaloid enriched fraction was significantly lower than vehicle.	(Loria et al., 2014)
	**Zembrin** extract, **mesembrine**, **mesembrenone** and **mesembrenol**	5-HT transporter binding assay and PDE4 inhibition (77 radioligand binding assays in broad receptor profiling)	Range of controls for each receptor	*In vitro* 750 μg/ml for Zembrin, 3 M for isolated compounds	Zembrin exhibited potent 5-HT binding (IC_50_ 4.3 g/ml) and PDE4 inhibition (IC_50_ 8.5 g/ml). The isolated alkaloid, mesembrine was the most active alkaloid against the 5-HT transporter (Ki 1.4 nM), whereas mesembrenone was active against the 5-HT transporter and PDE4 (IC50’s < 1 M). More than 50% inhibition was observed in the 5-HT transporter, GABA receptors, 2-opioid receptors, and cholecystokinin-1 targets.	(Harvey et al., 2011)
Anti-epileptic	**Zembrin** extract	Glutamate receptor assays (rat hippocampus) NMDA receptor, AMPA receptor, Metabotropic Glutamate Group I/II receptor, Metabotropic Glutamate Group III receptor	0.05 µM trans ACBD (NMDA agonist) - slight but significant decrease in signal, 0.10 µM (S)-(-)-5-fluorowillardine (AMPA agonist)- not able to exert action, 0.025 µM (±) trans ACPD (metabotropic glutamate receptor I/II agonist)—no change in signal, 0.05 µM O-Phospho-L-Serine (Metabotropic Glutamate Group III receptor agonist)- no change in signal	*ex vivo* 5 and 10 mg/kg Zembrin per day	Repetitive Zembrin administration resulted in mediation of AMPA and NMDA receptor associated with epileptic episodes.	(Dimpfel et al., 2018)
	**Mesembrine**, **Mesembranol**, **Mesembrenol** and **Mesembrenone**	Glutamate receptor assays - Artificial cerebrospinal fluid (ACSF)	0.10 µM (S)-(-)-5-Fluorowillardine	*In vitro* 8.65 nM Mesembrine, 17 nM for mesembranol, mesembrenol, and mesembrenone	Mesembrenol and mesembranol were able to prevent action of AMPA agonist associated with epileptic seizures.	(Dimpfel et al., 2018)
Anxiolytic	**Ethanolic extract** of **Zembrin (isolated compounds)**	Zebrafish model assay (Thigmotaxis and locomotor activity)	Diazepam (2.5, 5, 10 μM) exhibited best anxiolytic activity at 10 μM.	*In vivo* (extracts at 10, 15, 30, 50 μM)	Mesembrine, mesembranol, mesembrenol, and mesembrenone all demonstrated anxiolytic-like activity (50 μM illustrated highest activity).	(Maphanga et al., 2022)
	**Chloroform extract** of *S. tortuosum*	Chick anxiety-depression model	Imipramine (10 mg/kg), exhibited no effects in anxiety phase but increased DVoc in early and late phase depression state.	*In vivo* 10, 20, 30, 50, 75, 100 mg/kg	At concentrations of 10-30 mg/kg extract showed no effect on anxiety. Concentration of 75 mg/kg-100 mg/kg exhibited anxiolytic activity.	(Carpenter et al., 2016)
	Full **alkaloid extract**, the **alkaloid enriched fraction**, and **mesembrine**	Elevated plus maze assessment of anxiolytic activity	Chlordiazepoxide (5 mg/kg), spent more time on the open arms than vehicle control	*In vivo* 100, 20, and 20 mg/kg, respectively	No observed reduction in anxiety. None of the samples altered the time spent on open arms (none statistically significant)	(Loria et al., 2014)
	**Zembrin** extract	Pharmaco-fMRI (Perceptual-Load and Emotion-Matching Task) human trial (5-HT and PDE4)	Placebo capsule, only inert excipients	*In vivo*—clinical trial 25 mg	Zembrin resulted in a reduction in anxiety through reduction of amygdala reactivity in response to unattended facial fear.	(Terburg et al., 2013)
	**Methanol extract** of *S. tortuosum*	Elevated plus maze for psychological stress	0.85% sterile saline	*In vivo* 5 or 20 mg/kg/day of *S. tortuosum* extract for 17 days by daily oral gavage	Low doses (5 mg/kg/day) of extract showed marginal positive anxiolytic effects however both doses (5 and 20 mg/kg/day) illustrated negative side effects (inflammation and immune suppression).	(Smith, 2011)
Ataxia	Full **alkaloid extract**, the **alkaloid enriched fraction**, and **mesembrine**	Rotarod assessment of ataxia	Muscimol (2 mg/kg), fell off the drum significantly faster than vehicle treated group	*In vivo* 100, 20, and 20 mg/kg, respectively	The alkaloid-enriched fraction showed statistically lower times in assay and higher possibility of ataxia than mesembrine and full alkaloid extract.	(Loria et al., 2014)
Cognitive-enhancement	**Zembrin** extract	Rat pharmaco-EEG (Tele-Stereo-EEG)	0.9% NaCl (1 ml/kg), showed minor effects to delta and alpha-2 power in frontal cortex	*In vivo* 1.0, 2.5, 5.0, and 10.0 mg/kg	Comparable activity observed in Zembrin as seen in controls, Rolipram and Citicoline. Reduction in activity observed in dopaminergic and glutamatergic transmitter systems	(Dimpfel et al., 2018)
Neurodegenerative (Alzheimer’s dementia and Parkinson’s)	**Trimesemine** extract (TRI*)	Enzyme assays (MAO-A and AChE)	Galanthamine (0.0025 mg/ml, AChE), clorgyline (0.015 mg/ml, MAO-B)	*In vitro* 1.0, 0.1, 0.01, 0.001, and 0.0001 mg/m	TRI showed 30% inhibition against AChE at concentration of 2 mg/ml (IC_50_ AChE: unattainable, IC_50_ Galanthamine: 12.4 μg/ml). TRI showed 40% inhibition against MAO-B at concentration of 2 mg/ml (IC_50_ MAO-B: 408 μg/ml, IC_50_ clorgyline: 0.015 mg/ml)	(Coetzee et al., 2016)
Ergogenic^^4^^	**Zembrin** extract	Mood questionnaire a visual analog scales (VASs) – to assess fatigue and focus. Additionally, reactive performance assessments: multiple object tracking.	Not applicable	Not applicable	Significant improvement was observed in reactive performance in complex reactive task to improve cognitive load. No improvement in mood for sample group.	(Hoffman et al., 2020)

Bennett and Smith (2018) confirmed that a high-mesembrine *Sceletium* extract, Trimesemine™, could hold potential therapeutic activity in cytokine-induced depression, and they propose that the extract modulates the basal inflammatory cytokine profile whilst maintaining that there is no change in the acute response to pathogenic challenge. Furthermore, these findings illustrate a direct benefit to the attenuation of systemic low-grade inflammation in immune cells. This particular study did not test individual alkaloids, and such could not pinpoint the phytochemical constituent(s) responsible for the observed activity. In the future, chemical isolates may prove beneficial if the intention is to correlate bioactivity with specific alkaloid constituents so that our overall understanding of which phytochemicals hold bioactivity can be clarified.

Receptor screening of Zembrin^®^ (a standardised extract of *Sceletium tortuosum*) was conducted against 77 radioligand binding assays (0.75 mg/mL and a panel of phosphodiesterases) to compile a comprehensive list of potential CNS and other pharmacological targets (Harvey et al., 2011). The extract showed binding at the serotonin (5-HT) transporter, δ2- and μ-opioid receptors, the cholecystokinin-1 receptor, >80% inhibition at GABA receptors (non-selective), and PDEs 3 and 4 (Harvey et al., 2011). Some of the therapeutic applications of these targets are emesis, obesity, anxiety, and migraine linked to the serotonin (5-HT) transporter (Pithadia and Jain, 2009). The δ2- and μ-opioid receptors are involved in maintaining epileptic seizure, emotional responses, immune function, obesity, cell proliferation, respiratory and cardiovascular control, and several neurodegenerative disorders (Feng et al., 2012). The cholecystokinin-1 receptor is involved in gastrointestinal and metabolic diseases (Berna and Jensen, 2007), whereas GABA receptors are involved in pathologies ranging from epilepsy, schizophrenia, anxiety disorders, and premenstrual dysphoric disorder (Wong et al., 2003). The PDE3 receptor is responsible for platelet activation/aggregation (Beca et al., 2011) and vascular smooth muscle proliferation (Beca et al., 2011; Begum et al., 2011), whereas the PDE4 receptor is linked to inflammatory conditions including asthma, chronic obstructive pulmonary disease (COPD), psoriasis, atopic dermatitis (AD), inflammatory bowel diseases (IBD), rheumatic arthritis (RA), lupus, and neuroinflammation (Li et al., 2018). This report only presented findings on the affinity of Zembrin^®^ to different receptors. Further studies would need to be investigated for activity against these specific pathologies. Plant extracts have numerous metabolites that work in synergy to affect their biological influence, and it is thus possible that multiple metabolites are potentiating the mood-elevated activity aside from mesembrine alone, as suggested by Lubbe et al. (2010). The Harvey et al. (2011) study supports ethnobotanical use as a mood elevator by the observed serotonin transport activity in response to Zembrin^®^. The serotonin receptor influences a myriad of biological and neurological processes such as anxiety, appetite, aggression, and depression (Mück-Šeler and Pivac, 2011; Zhang and Stackman, 2015). Evidence of anxiolytic effects of *Sceletium* in humans (Gericke and Viljoen, 2008) has partially been supported in a study using a rat model of restraint induced stress (Smith, 2011). The binding of compounds to various sites on the 5-HT transporter (SERT) is considered evidence of potential serotonin reuptake inhibition, a common target of antidepressant drugs. A selection of alkaloids, mesembrine, mesembrenone, and mesembrenol, from *Sceletium tortuosum*, were tested for their affinity for SERT with *K*i’s of 1.4 nM, 27 nM, and 63 nM, respectively (Harvey et al., 2011). These values were significantly higher than other alkaloids, such as buphanidrine or distichamine, isolated from Amaryllidaceae, with reported *K*i’s of 312 μM and 868 μM, respectively (Neergaard et al., 2009). These compounds have been found to already possess well-established antidepressant activity, found in *Boophone disticha* (L.f.) Herb (Amaryllidaceae) (Neergaard et al., 2009). There is some evidence that argues against *Sceletium* purely acting as a selective serotonin reuptake inhibitor (SSRI), as repeated administration of SSRIs has been linked to hyposensitivity to SSRIs, as a result of an upregulation in PDE4 (Ye et al., 2000). However, it has been demonstrated that PDE4 activity decreased after *Sceletium* administration (Harvey et al., 2011). The Harvey et al. (2011) study was the only one testing a *S. tortuosum* extract on a number of receptors. Although the authors’ study looked at the pharmacokinetics of individual compounds against each receptor, it may be valuable to examine the extracts with varying concentrations of compounds as a means to assess samples in a context more aligned with ethnobotanical use.

*In vivo* testing of *Sceletium* alkaloids has been performed using rat models designed for mental disorders such as neurodegeneration, like Alzheimer’s disease (AD), epilepsy, and depression (Loria et al., 2014) (**Figure 6C**). Loria et al. (2014) found that mesembrine from *S. tortuosum* had analgesic and antidepressant activity. *Sceletium* species have exhibited potential therapeutic activity *in vivo* using rodent models for AD, anxiety, and depression (Gericke and Viljoen, 2008; Krstenansky, 2017). A summary of the CNS-related activity, together with the recent anti-inflammatory activity of *Sceletium*, is presented in **Table 4**. With the PDE4 activity of *Sceletium* extracts noted by Harvey et al. (2011) and new *in vivo* on the receptor itself, there is evidence suggesting that inhibitors from *Sceletium* can aid to reverse depression, improve cognitive ability, and reduce anxious states. The anxiolytic activity of *Sceletium* may be attributed to other mechanisms in addition to serotonin-reuptake inhibition such as monoamine release (Coetzee et al., 2016). *In vivo* testing of *Sceletium* alkaloids has been performed using rat models designed for mental disorders such as neurodegeneration, like Alzheimer’s disease (AD), epilepsy, and depression (Loria et al., 2014) (**Figure 6C**). Loria et al. (2014) found that mesembrine from *S. tortuosum* had analgesic and antidepressant activity. *Sceletium* species have exhibited potential therapeutic activity *in vivo* using rodent models for AD, anxiety, and depression (Gericke and Viljoen, 2008; Krstenansky, 2017). A new structure–function relationship for *Sceletium* alkaloids was suggested by Timoneda et al. (2019); tests performed on rats using Zembrin^®^ found new evidence of electric excitability of the rat hippocampus supporting this new relationship.

**Figure 12:**
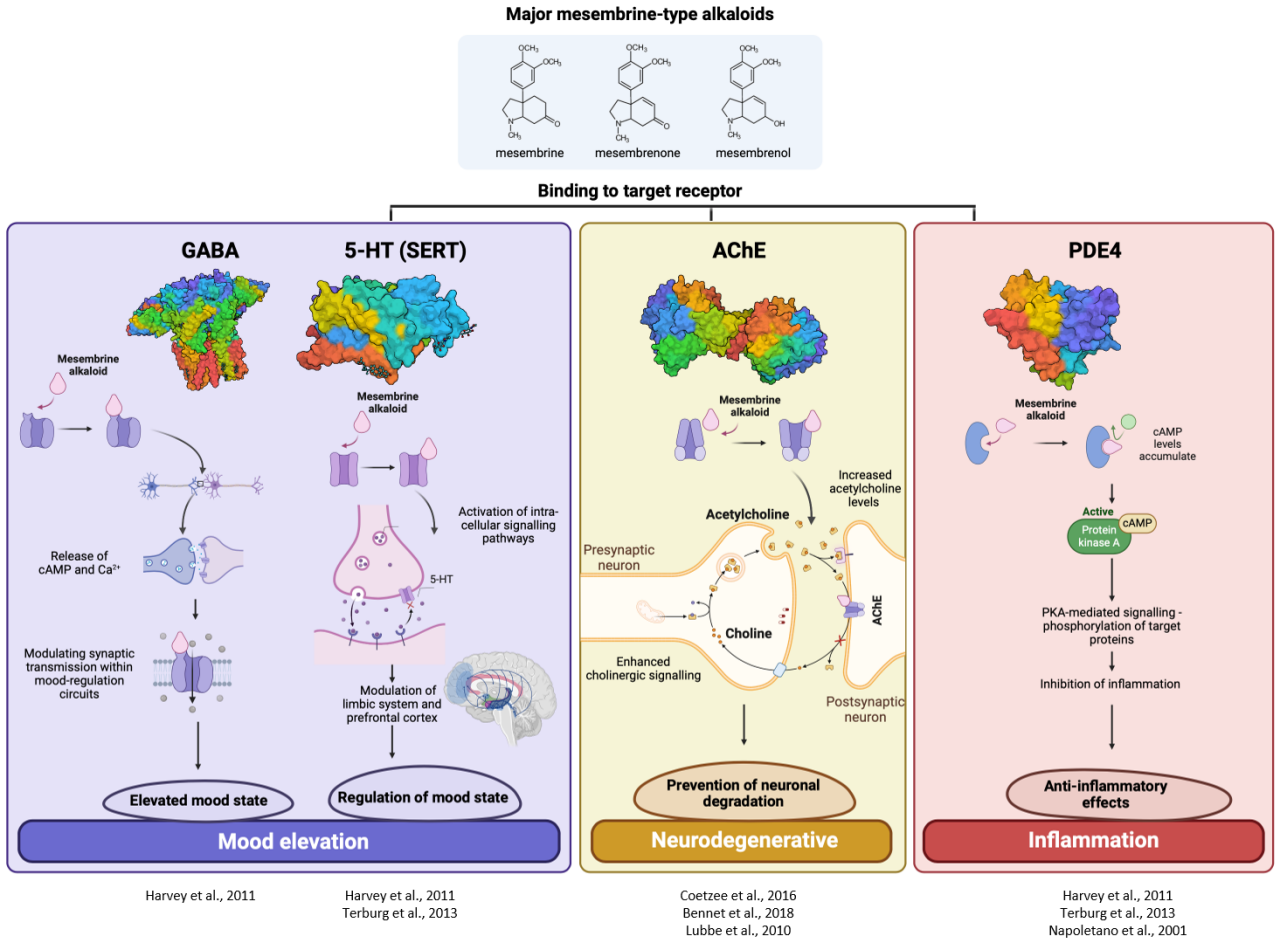
Summary of the mechanistic actions reported in literature for *Sceletium* mesembrine metabolites acting on targets regulating mood elevation (GABA and 5-HT), neurodegenerative disease (AChE), and inflammation (PDE4).

The summarised findings of the proposed molecular mechanisms related to the mood elevation and neuroprotective and anti-inflammatory activity of *Sceletium* is presented in **Figure 12**. The exact mechanisms of action in the case of *Sceletium* and its alkaloidal metabolites are largely unknown; however, some receptor-based *in vivo* and clinical trials have been performed.

Mesembrenone has shown anti-tumour activity against a murine non-tumoral fibroblast cell line and a human tumoral cell line (Molt4), and Weniger et al. (1995) tested 25 alkaloids from Amaryllidaceae with only mesembrenone showing some specificity for Molt4 cells. Extracts high in mesembrine and Δ^7^ mesembrenone were shown to exert anti-inflammatory and antioxidant activities *in vitro*, respectively (Bennett et al., 2018). The mesembrine-rich extract, which was less refined as compared with the Δ^7^ mesembrenone extract, exhibited broad dose range efficacy and may serve as a promising therapeutic in the setting for chronic diseases, being safe when administered at low doses. Due to the aetiology of both diabetes and obesity being linked to inflammation and excess glucocorticoid production, these findings may hold value in chronic lifestyle disease management such as diabetes types 1 and 2. For such data to be translated into a pharmaceutical drug discovery chain, endocrine-immune (IL-6 and MCP-1) interactions need to be investigated.

The potential therapeutic activity of mesembrine alkaloids toward inflammatory diseases ranging from asthma, chronic obstructive pulmonary disease, psoriasis, and treating depression has been investigated (Houslay et al., 2005). The anti-inflammatory activity is suspected to be due to the activity of mesembrine-HCl acting as an inhibitor of phosphodiesterase-4 (PDE4), with observed activity at an IC_50_ of 29 µM (Napoletano et al., 2001). The selective inhibition of the PDE4 family of enzymes is predicted to generate great functional effects, as evidenced by PDE4 enzymes being a major therapeutic target for inflammatory diseases (Gericke and Viljoen, 2008). PDE4 has also been identified to play a role in the inflammatory system (Banner and Trevethick, 2004; Dastidar et al., 2007; Li et al., 2018). Harvey et al. (2011) found that the alkaloids mesembrenol, mesembrine, and mesembrenone inhibited PDE4B with IC_50_ values of 16, 7.8, and 0.47 μM, respectively. The positive control, rolipram, had an IC_50_ for PDE4B of 0.13 μM (MacKenzie and Houslay, 2000). Another enquiry illustrated the activity of Zembrin^®^ *in vivo* to correlate this mechanism of action observed *in vitro* in freely moving rats interpreted as an electopharmacogram^5^ (Dimpfel et al., 2016).

More recent studies include the work of Reay et al. (2020), where the anxiolytic properties of Zembrin^®^ were assessed in a double-blind, placebo-controlled behavioural study with healthy human volunteers. A dose of 25 mg was administered, and stress was assessed amongst young human adults using multitasking and simulated public speaking frameworks. The study failed to replicate previously reported enhancement of cognitive function, and this was the first evidence of Zembrin^®^ having no impact on non-executive memory processing in healthy participants.

Maphanga et al. (2020) assessed the anxiolytic activity in a zebrafish behavioural assay for a number of medicinal plants, one of which was *S. tortuosum*. Additionally, no toxic effects were observed on the zebrafish in the assay. The model proves to be an appropriate and repeatable assay for assessing the anxiolytic activity of *Sceletium* extracts. Supporting this work is the rising application of the zebrafish animal model as it is claimed to be adequately comparable with humans, sharing approximately 70%–80% genetic homology with humans (Barbazuk et al., 2000; Goldsmith, 2004). The ergogenic effect of a *S. tortuosum* supplement was studied using men and women for 8 days (Hoffman et al., 2020), but no benefits in mood were observed. However, there were significant improvements noted in complex reactive performance tasks that include the stress of cognitive load. Other forms of assessments linked to measuring cognitive and mood information may yield different results. Furthermore, no pharmacokinetics and absorption data were presented in the study.

Maphanga et al. (2022) assessed isolated alkaloids from the extract described in Shikanga et al. (2011), at concentration ranges of 10 μM, 15 μM, 30 μM, and 50 μM with the greatest activity across alkaloids observed at 50 μM, using a zebrafish assay that focused on toxicity, measured as MTCs (maximum tolerated concentrations), where locomotor activity was above 50 μM. The study of Gericke et al. (2022) reported that Zembrin at doses of 25 mg/kg and 50 mg/kg was effective as an antidepressant in the forced swim test (FST) and performed better than the control (escitalopram). This was the first study to date that compared Zembrin with an SSRI in a rodent model of this kind, supporting the therapeutic use of *S. tortuosum* for mood disorders.

Bioavailability studies on *Sceletium* and its alkaloids are greatly lacking in research. Shikanga et al. (2012a) presented findings on the permeability of mesembrine across the buccal, intestinal, and sublingual mucosal membranes. In that study, mesembrine had a higher permeability across intestinal tissue than the positive control caffeine but the permeability was lower in the buccal and mucosal sublingual membranes. Manda et al. (2017) showed that the oral bioavailability of mesembrine and mesembrenone in mouse plasma (using UHPLC‐QToF‐MS) was poor and below the detection limit. Bioavailability information regarding other alkaloids and chemotypes from *Sceletium* is still not documented in terms of data on the permeability of these alkaloids across buccal, intestinal, and sublingual mucosal tissues. It is thus imperative that more attention should be placed on such to provide new evidence linked to bioavailability in order to support or refute ethnobotanical claims. It may also be of interest to monitor cultivated and commercially available samples for pesticide residues and toxic alkaloids in other plants that may have mistakenly been gathered during the harvesting of wild populations of *S. tortuosum*, as this species is often found under the canopy of other small shrubs in the wild and in close association with a diverse range of other species. At this present time, there is no information in this respect and the monitoring of plant or chemical contaminants is thus urgently needed.

### Biological associations

Recently, a new avenue of investigation was based on investigating the association of endophytic fungal communities on *S. tortuosum* (Manganyi et al., 2018) (**Table 5**). *Fusarium, Aspergillus*, and *Penicillium* were amongst the fungal endophytes found in the plant. In total, there were 60 endophytic fungal species successfully isolated and identified, belonging to 16 genera. The antibacterial activity of this endophytic fungi was also investigated, where it was found that some fungal isolates could provide sources of novel antimicrobial agents against antibiotic-resistant strains (Manganyi et al., 2019). This is also the first investigation to report on secondary metabolites from endophytic fungi, *F. oxysporum* (GG 008, accession no. KJ774041.1), isolated from *S. tortuosum* (Manganyi et al., 2019).

**Table 5:** Other notable biological activity of *Sceletium tortuosum* extracts and compounds (*Note that TRI is an extract of a *S. tortuosum* and *S. expansum* hybrid).

Effect	compound/ extract	Model/Target	Positive control	Formulation/dosage	Result/mechanism/method	Reference
Anti-bacterial	Secondary metabolites from endophytic fungi on *S. tortuosum* (tested against Gram-positive and Gram-negative bacteria)	Disc diffusion assay	*Enterococcus gallinarum*	*in vitro* 1 × 10^7^ cells/mL of bacteria suspensions for each isolate	*Fusarium oxysporum* displayed antibacterial activity, linked to high levels of 5-hydroxymethylfurfural (HMF) and octadecanoic acid. Narrow spectrum of activity observed in 15% of the fungal extracts.	(Manganyi et al., 2019)
Anti-HIV	*S. tortuosum* extract	Inhibition activity against HIV-1 enzymes; HIV-1 protease assay, HIV-1 reverse transcriptase colorimetric assay and HIV-1 Integrase colorimetric assay	10 μg/mL Acetyl pepstatin (92.6%), 25 μg/mL Doxorubicin (90.1% inhibition), Sodium azide	*in vitro* 25, 50, 100, 150, 200 and 250 μg/mL (Protease assay). 25, 50, 100, 150, 200, 250 μg/mL (reverse transcriptase assay). 0.2, 0.4, 0.8, 1 and 2 mg/mL (integrase assay)	Inhibition of protease (PR) and HIV-1 reverse transcriptase (RT) by ethyl acetate and ethanol extracts, respectively.	(Kapewangolo et al., 2016)
Anti-inflammatory	*S. tortuosum* extract (two extraction method samples)	Human astrocyte viability assay	20 μg/ml *Escherichia coli* lipopolysaccharide (LPS)	*ex vivo* 1 mg/ml extract A and 3.7 mg/ml extract B	Mesembrine-rich extract showed cytoprotetive and anti-inflammatory activity. Extract B showed notable activity in improving the capacity of reductive capacity in basal mitochondria.	(Bennett et al., 2018)
Anti-oxidant	*S. tortuosum* extract (two extraction method samples)	DPPH inhibition	Ascorbic acid	*in vitro* 1 mg/ml extract A and 3.7 mg/ml extract B	Extract B showed good activity in that the total phenolic content was 20 times higher than A and performed comparably with the positive control (ascorbic acid)	(Bennett et al., 2018)
Bioavailability (Mucosal transport)	Plant extract (MeOH, water, Acid extract, pure alkaloids)	Intestinal, buccal and sublingual transport studies	40 µg/mL Caffeine	*in vitro* Mesembrenone: 90 µg/mL, Mesembrenol: 80 µg/mL, Mesembrine: 40 µg/mL, and mesembranol: 40 µg/mL (Pure extracts). Plant extract at 40 µg/mL)	The water extract illustrated best permeability. Overall alkaloids from *S.* tortuosum showed relatively good permeability across sublingual mucosal tissue and poor permeability across buccal tissue.	(Shikanga et al., 2012a)
Immunomodulatory	Trimesemine™ (TRI*)	Primary human monocyte viability	1 mg/ml LPS	*in vitro* 0.01 mg/ml or 1 mg/ml TRI extract	An up-regulation of monocyte IL-10 secretion illustrated anti-inflammatory activity of TRI at a basal level. No cytotoxic effects noted.	(Smith, 2018)
Neuro-protection	*S. tortuosum* extract (two extraction method samples)	Enzyme assays, AChE and tyrosinase	Galantamine and kojic acid	*in vitro* 1 mg/ml extract A and 3.7 mg/ml extract B	Extract A illustrated mild inhibitory effects (IC_50_ – 1.621 ± 0.75) while extract B acted as a potent inhibitor (IC_50_ – 0.5908 ± 0.01).).	(Bennett et al., 2018)
Stress and hypertension	Zembrin extract	Randomised, double-blind, parallel-group, placebo-controlled single centre study	D4-cortisol (15 ng)	*in vivo* Clinical trial. 8mg extract Sceletium tortuosum (Zembrin), 25 mg extract Sceletium tortuosum (Zembrin), and placebo treatment	The apparent difference in vital signs over duration of screening period (3 months). Doses were well tolerated with reported improvements in anxiety in sleep.	(Swart and Smith, 2016)
Safety and tolerance	Trimesemine™ (TRI)	Steroid levels in human adrenocortical carcinoma cells (H295R - steroidogenesis).	10 μM Forskolin, increased steroid production significantly (2.3-fold) in all pathways	*in vitro* 1 mg/ml and 1 μg/ml	Inhibition in androstenedione and testosterone production across all doses was observed. The highest dose of TRI (1 mg/ml, 34.5 μM mesembrine) decreased 16-hydroxyprogesterone levels…	(Nell et al., 2013)
Toxicity and sub-chronic toxicity	Zembrin extract	Repeated dose oral toxicity in rats	Vehicle control	*in vivo* 0 (vehicle-control), 250, 750, 2500 and 5000 mg/kg bw/day by gavage (14-day study) and f 0 (vehicle-control), 100, 300, 450 and 600 mg/kg bw/day by gavage (90-day study)	Within populations of Crl:(WI)BR Wistar rats, irrespective of gender. No adverse effects were noted at a dose of 600 mg/kg bw/day in a 14- and 90-day study.	(Murbach et al., 2014)

### Plant propagation techniques

Faber et al. (2020) reported on the influence of soilless growth medium (pure silica sand, 50% silica sand with 50% coco peat, 50% silica sand with 50% vermiculite, and 50% silica sand with 50% perlite) and fertigation regimes (nutrient solution administered in intervals from 1 to 5 weeks) on shoot and root growth as well as how these factors influenced alkaloid levels (Δ^7^-mesembrenone and mesembrine). Higher mesembrine levels were detected in the shoots whereas roots had higher concentrations of mesembrenone and Δ^7^-mesembrenone. The major observation is that the influx of secondary metabolites in *S. tortuosum* seems to possibly respond to biotic and abiotic factors (Bourgaud et al., 2001; Ashraf et al., 2018).

To date, there have only been three studies investigating the micropropagation of the medicinally important, *S. tortuosum* (Sreekissoon et al., 2021a, 2021b; Makunga et al., 2022). The illegal harvesting and exploitation of *S. tortuosum*, due to the demand as a recreational drug linked to its euphoric properties, is a driving factor for the dire need for the development of micropropagation techniques. This approach could offer a direct and standardised source of mesembrine alkaloids. Sreekissoon et al. (2021b) investigated whether *in vitro* regeneration of micropropagules with auxins could be acclimated *ex vitro*. Sreekissoon et al. (2021a) confirm how smoke water influences the germination, seedling vigour and growth of *S. tortuosum* when it is applied at ratio of 1:1,000. A limitation of these studies was that key biomarker compounds were not monitored in the microplant regenerates. Makunga et al. (2022) generated different *in vitro* morphotypes and showed for the first time that Δ4-mesembrenone, mesembrenol, mesembrine, and mesembranol accumulate in micropropagated plants and callus. The utilisation of micropropagation using the dehydrating and rehydration technique outlined in this report resulted in *in vitro* mesembrine accumulation comparable with wild-type material collected (Zhao et al., 2018).

### Legislation, toxicology, and safety of *Sceletium* alkaloids

Toxicological assessments on *Sceletium* are limited with the first formal *in vivo* toxicological assay being performed by Murbach et al. (2014) on the mesembrine-rich extract Zembrin^®^ (**Table 5**). They found that Zembrin^®^, in male and female Crl:(WI)BR Wistar rats, showed no mortality or treatment-related adverse effects spanning 14 or 90 days with doses of 600 mg/kg bw/day and 5,000 mg/kg bw/day, respectively (Murbach et al., 2014). A greater effort in understanding cytotoxic effects of *Sceletium*-derived extracts and their potential drug–herb interactions is also urgently needed. However, Brendler et al. (2021) indicated that there are no drug–herb interactions that are currently known, although these authors indicate for wise use of serotonin uptake or release medications that may be prescribed for psychiatric conditions.

Clinical administration of *S. tortuosum* has been carried out by Gericke (2001). The clinical case study reported on three individuals who have been prescribed *S. tortuosum* in tablet form. Patients were being treated for anxiety and depression with one patient having been diagnosed with a personality disorder (dysthymia). In the latter case, the patient described an overall decrease in anxiety and was more able to cope with stress in her life. There were no apparent withdrawal symptoms for all three individuals once they stopped taking the treatment.

The plant is often marketed as a food supplement in the South African natural products sector and not as a scheduled drug. According to Brendler et al. (2021), regulation of herbal medicinal products is highly complex and heterogenous because it is linked to country-specific legislation that governs the use of natural products in different parts of the world. These authors provided a comprehensive and detailed discussion with regard to regulatory legislation that governs the use of *Sceletium* for human consumption. Some of the key points incorporated in that paper are summarised briefly below.

In Europe, at present, *Sceletium*-based products have not been legally approved, but in Russia, specifically, such products fall under the category of scheduled pharmaceutical drugs. Zembrin®, a standardised extract, has had a ‘generally recognised as safe’ (GRAS) status in the United States of America since 2011, allowing for its inclusion in dietary supplements. On the other hand, in Canada, natural products with *Sceletium* have been approved since 2014 and a wide range of different products are thus available to consumers. Brendler et al. (2021) advocate for an intensive push to research, not only Zembrin® but also other standardised extracts in clinical settings to circumvent regulatory and legislative barriers that may be restricting a wider acceptance and entry of *Sceletium* products into global markets. The United Nations Office on Drugs and Crime flagged *Sceletium* as a plant of concern when reporting on substances of concern in 2013 as part of a report on the obstacles in the identification and regulation of new psychoactive substances (UNODC, 2013). The nature of *Sceletium* being classified as herbal or dietary supplements often exempts it from mandatory testing by the US Food and Drug Administration (FDA). Thus, without adequate quality control and authentication systems in place, herbal product producers can adulterate samples with other stimulants such as ephedrine (which has been banned in herbal products and supplements in Germany) (Lesiak et al., 2016).

Dietary supplementation is popular for those that partake in sports recreationally or as professional athletes, and sports performance-enhancing natural products are thus highly sought after. Legislation around the utilisation of *S. tortuosum* extracts for elite athletes in competitive sports as a dietary supplement hangs in the balance as some regulatory bodies have denoted its status under the categories of ‘unauthorised novel food’, and it is included also in the European Food Safety Authority Compendium of Botanicals as concerning for human consumption (Jędrejko et al., 2021). Due to its effects on brain function and cognition, it has not necessarily been approved for routine use by the World Anti-Doping Agency (WADA) which regulates permissible dietary supplements for athletes.

## Conclusions and future perspectives

The bibliometric analysis showed that South Africa has established a strong network with researchers working on *Sceletium* and its medicinal value, but there is an apparent lack of synergy and coordination between research groups located in the native land of this genus. The reason for this is not obvious but could be linked to historical networks and collaborations being preferred and strong competition for limited funding between research groups. A higher degree of collaboration is thus foreseen to encourage greater progress in transforming latent botanical assets into consumer products.

A portion of the current review has highlighted the development of quality control tools for commercial and wild-harvested *Sceletium* species, with the focus being on *S. tortuosum* due to its rising commercialisation status in global markets. With the popularity of *Sceletium* growing as a recreational natural product, a more diverse range of products emanating from a growing number of manufacturers is an imminent probability. However, the expansion of the industry may bring about an increased frequency of herb–drug adulterations as seen with many other natural products that are popular in commercial settings (Campbell et al., 2013; Seethapathy et al., 2015; Booker et al., 2016). There is thus a critical urgency that is required in the research and development of analytical techniques and protocols that are rapid, robust, and reproducible, which may be highly efficacious in their detection capacity for adulterants that may occur in products even at minute scales. Such techniques need to be able to have high resolving power and identify adulterants that would otherwise not be identified due to their similarity in polarity with some of the biomarker compounds. From a practical perspective, these instruments are not always common in laboratories and they are expensive, requiring users to have specialised training and highly sophisticated scientific expertise.

The choice of chemo-elite types that can become easily domesticated may assist the production of quality-assured natural products, generating an industry that will gain consumer trust. This is viewed as being of high importance when considering that some *S. tortuosum* wild types may produce mesembrine alkaloids at exceedingly low concentrations and other *Sceletium* spp. show a complete lack of the key biomarker compounds that are routinely examined by the phytopharmaceutical and nutraceutical industries (Patnala and Kanfer, 2013). Because the different species are similar to each other, this makes them highly vulnerable to misidentification and incorrect identification and unregulated collection of these plants may set in motion their overharvesting thereby creating serious conservation concerns of the genus. The number of studies looking into genetic approaches for quality control tools is remarkably absent in *Sceletium* research. DNA fingerprinting and biomarker identification (Klak et al., 2007; Shikanga et al., 2013; Zhao et al., 2018), single-nucleotide polymorphisms (SNPs), and microsatellite loci (nuclear short sequence repeats, SSR) have been commonly used for genetic-based quality control (Laurie et al., 2010). The absence of quality pure chemical standards has greatly hindered the absolute quantification of the mesembrine alkaloids, and more effort is thus required to fill this gap as the lack of reference compounds makes the identification and profiling of both minor and major alkaloids synthesised by *S. tortuosum* and its relatives more challenging.

Historical ethnobotanical records allude to a practice of fermentation of *S. tortuosum* when it is used by local indigenous people, but scientific evidence of the fermentation on phytochemicals of the plants remains in contention as current fermentation studies do not correlate with each other. Studies investigating the manipulation of biosynthetic pathways in combination with fermentation studies may thus prove valuable in deepening the general understanding of the effects of the fermentation treatment(s) and its biological effects in animal systems. Despite this, the pharmacological tests, whether they be in *in vitro* and/or *in vivo* experiments, have increasingly supported the traditional use of *S. tortuosum* as a mood-elevator and anxiolytic agent. However, its anti-inflammatory and immunomodulatory effects have been insufficiently investigated up until recently. At this point, there has been some evidence that highlights the beneficial physiological effects of *S. tortuosum* extracts as a plant medicine, which extend beyond its psychoactive effects, with potential therapeutic activity targeted at diabetes and obesity (Bennett and Smith, 2018). Interest in developing *Sceletium* into an additive for foods, beverages, and supplements aiding in depressive and anxiolytic disorders has been happening for over a decade (Gericke and Viljoen, 2008), with some products finding the market. If this is to be fully realised, a fundamental field of investigation that will need to be looked into is the standardised cultivation of the plants for their phytochemicals. This can be achieved through the manipulation of secondary metabolites using physiological stress such as light, pH, and nutrient stress in *Sceletium* species, which is currently a void in the research scope.

To enable such studies, a reference genome is also urgently needed for *S. tortuosum* as currently the genetic resources that may assist with understanding the genetic and biochemical controls that are involved in the biosynthetic pathways of mesembrine alkaloids are unavailable. Such resources would thus provide additional research efforts into the control of metabolic flux linked to mesembrine biosynthetic pathways and identify regulatory promoters that influence the synthesis of the unique alkaloids of *Sceletium*. Systems biology studies using a multiomics approach may further assist with the full characterisation of pathway interactions that may lead to a better understanding of the metabolic networks that control alkaloid biosynthesis routes of *Sceletium tortuosum* and related sister species.

Ultimately, *Sceletium* and its alkaloids hold great potential in future endeavours and might provide novel insights into the synthesis pathways of *Sceletium*-specific alkaloids and their genetic regulatory controls whilst studies on inflammation activity and its phytomedicinal applications rise in industry.

## Data availability statement

The original contributions presented in the study are included in the article/supplementary material. Further inquiries can be directed to the corresponding author.

## Author contributions

KR: Data curation, Formal analysis, Investigation, Methodology, Software, Validation, Visualization, Writing – original draft. GS: Conceptualization, Supervision, Writing – review & editing. NM: Conceptualization, Funding acquisition, Project administration, Resources, Supervision, Writing – review & editing.

## Footnotes

1 LE: liquid solvent extraction.

2 N/A: as study directly analysed samples in the inlet source.

3 Distress vocalizations in an avian model.

4 Increase in capacity for mental strain and reduction in fatigue.

5 An analysis recording the field potentials in the frontal cortex, striatum, hippocampus and reticular formation while administered drugs.
